# Beyond Traditional Morphological Characterization of Lung Neuroendocrine Neoplasms: In Silico Study of Next-Generation Sequencing Mutations Analysis across the Four World Health Organization Defined Groups

**DOI:** 10.3390/cancers12102753

**Published:** 2020-09-24

**Authors:** Giovanni Centonze, Davide Biganzoli, Natalie Prinzi, Sara Pusceddu, Alessandro Mangogna, Elena Tamborini, Federica Perrone, Adele Busico, Vincenzo Lagano, Laura Cattaneo, Gabriella Sozzi, Luca Roz, Elia Biganzoli, Massimo Milione

**Affiliations:** 1 1st Pathology Division, Department of Pathology and Laboratory Medicine, Fondazione IRCCS (Scientific Institute for Research, Hospitalization and Healthcare) Istituto Nazionale dei Tumori, via Venezian 1, 20133 Milan, Italy; giovanni.centonze@istitutotumori.mi.it (G.C.); vincenzo.lagano@istitutotumori.mi.it (V.L.); laura.cattaneo@istitutotumori.mi.it (L.C.); 2 Tumor Genomics Unit, Department of Research, Fondazione IRCCS Istituto Nazionale dei Tumori, via Venezian 1, 20133 Milan, Italy; gabriella.sozzi@istitutotumori.mi.it (G.S.); luca.roz@istitutotumori.mi.it (L.R.); 3 Molecular Biotechnology and Bioinformatics, Department of Biosciences, University of Milan, Via Festa del Perdono 7, 20122 Milan, Italy; davide.biganzoli1@studenti.unimi.it; 4 Medical Oncology Department, Fondazione IRCCS—Istituto Nazionale dei Tumori, via Venezian 1, 20133 Milan, Italy; natalie.prinzi@istitutotumori.mi.it (N.P.); sara.pusceddu@istitutotumori.mi.it (S.P.); 5 Institute for Maternal and Child Health—IRCCS Burlo Garofolo, Via dell’Istria 65, 34137 Trieste, Italy; alessandro.mangogna@burlo.trieste.it; 6 2nd Pathology Division, Department of Pathology and Laboratory Medicine, Fondazione IRCCS—Istituto Nazionale dei Tumori, via Venezian 1, 20133 Milan, Italy; elena.tamborini@istitutotumori.mi.it (E.T.); federica.perrone@istitutotumori.mi.it (F.P.); adele.busico@istitutotumori.mi.it (A.B.); 7 Unit of Medical Statistics, Biometry and Bioinformatics “Giulio A. Maccacaro”, Campus Cascina Rosa, Fondazione IRCCS Istituto Nazionale Tumori, via Venezian 1, 20133 Milan, Italy; elia.biganzoli@unimi.it; 8 Department of Clinical Sciences and Community Health, Laboratory of Medical Statistics, Biometry and Epidemiology “G.A. Maccacaro”, Data Science Research Center, University of Milan, Via Augusto Vanzetti 5, 20133 Milan, Italy

**Keywords:** lung cancer, lung neuroendocrine neoplasm, in silico analysis

## Abstract

**Simple Summary:**

Lung neuroendocrine neoplasms (LNENs) classes, as proposed by the World Health Organization 2015, do not provide properly prognostic and therapeutic indications. In fact, high-throughput molecular analysis, based on next-generation sequencing, identified novel molecular subgroups, associated with different genomic signatures, that could pave the way for alternative therapeutic approaches. The present review, coupled with in silico molecular analysis, could show the current genomic alterations state in actual LNENS groups. Interestingly our manuscript suggests that the molecular novelties could improve the LNENs therapeutics efficacy. In more detail, we reported the differences of gene alterations and mutational rate between LNENS, confirming the central pathogenetic role given by a different mutational rate in chromatin remodeling genes and tumor suppressors TP53-RB1. In conclusion, our results underlined that a further molecular layer is needed to improve the efficacy of LNENs medical treatment.

**Abstract:**

Lung neuroendocrine neoplasms (LNENs) represent a rare and heterogeneous population of lung tumors. LNENs incidence rate has increased dramatically over the past 30 years. The current World Health Organization LNENs classification (WHO 2015), distinguished four LNENs prognostic categories, according to their morphology, necrosis amount and mitotic count: typical carcinoid (TC), atypical-carcinoid (AC), large cell neuroendocrine carcinoma (LCNEC) and small cell lung cancer (SCLC). At present, due to their rarity and biological heterogeneity there is still no consensus on the best therapeutic approach. Next-generation-sequencing analysis showed that WHO 2015 LNENs classes, could be characterized also by specific molecular alterations: frequently mutated genes involving chromatin remodeling and generally characterized by low mutational burden (MB) are frequently detected in both TC and AC; otherwise, *TP53* and *RB1* tumor suppressor genes alterations and high MB are usually detected in LCNEC and SCLC. We provide an overview concerning gene mutations in each WHO 2015 LNENs class in order to report the current LNENs mutational status as potential tool to better understand their clinical outcome and to drive medical treatment.

## 1. Introduction

Lung neuroendocrine neoplasms (LNENs), are heterogeneous neoplasms originating in the bronchial tract covering 20–25% of all lung cancer [1,2]. LNENs incidence rate tumors has risen over the past 30 years [3,4,5,6]. According to the World Health Organization (WHO) 2015, LNENs are classified into four subtypes according to morphological features, necrosis amount and mitotic count: typical carcinoid (TC), well differentiated, mitotic index (MI) < 2 mitoses/2 mm^2^ and absence of necrosis; atypical carcinoid (AC): well differentiated, 2 < MI < 10 mitoses/2 mm^2^ and necrosis; large cell neuroendocrine carcinoma (LCNEC): poorly differentiated, constituted by neoplastic cells with abundant cytoplasm, extensive/geographic necrosis and prominent nucleoli, MI > 10 mitoses/2 mm^2^ [1]; small cell lung carcinoma (SCLC): poorly differentiated, MI > 10 mitoses/2 mm^2^ and diffuse necrosis. WHO 2015 LNENs classes show different clinical outcome: TCs good prognosis, ACs intermediate prognosis due to high risk of metastasis, LCNEC and SCLC aggressive clinical course and poor prognosis [1,2,7].

Based on histology and clinical behavior, TCs and ACs can be grouped into well differentiated neuroendocrine tumors (NETs), while LCNECs and SCLCs into poorly differentiated carcinomas (NECs) [8]. Therefore, the Agency for Research on Cancer (IARC) and WHO recently proposed NETs and NECs as new uniform classification for all NENs across different sites, supported by morphological, clinical, histological, epidemiologic, prognostic and genetic differences at specific anatomic sites [9].

Molecular studies on LNENs showed that: (i) NETs (TC and AC) are characterized by low mutational burden and that *MEN1* and *ARID1A* (chromatin remodeling and histone modification) plays a pivotal role in NETs pathogenesis [10,11]; (ii) NECs showed high mutational burden, with a strong relationship with smoking, and that *TP53* and *RB1* drive their pathogenesis [10,12,13,14].

At present, LNENs clinical management is strongly based on accurate diagnosis.

According to ENETS guidelines [15], surgery remains the backbone for NETs in early stages, while, for advanced and metastatic diseases, a multimodal approach, including surgery and systemic treatments, is requested [15,16,17,18]. Somatostatin analogues (SSAs), target therapies (everolimus) and peptide receptor radionuclide therapy (PRRT) are the most commonly used strategies for NETs in advanced stages [19,20,21,22]. For patients with NECs, often metastatic at the diagnosis, chemotherapy and immunotherapy represent the most important therapeutic options. Surgery in LCNECs has the same indications of non-small cell lung cancer (NSCLC), while in SCLCs is to be considered only in the very early stages [19,20,21,22]. However, especially for ACs and LCNECs, considering the rarity and the biological heterogeneity of these tumors, there is no global consensus on best therapeutic approach.

High-throughput analysis with next-generation sequencing (NGS) allowed a stratification of the current four histological variants and the identification of new molecular subgroups with different genomic signatures [12,23,24,25]. These studies mainly concern ACs and LCNECs, highlighting a strong molecular and biologically heterogeneity, allowing a better prognostic stratification and predicting the therapy outcome [10,26,27]. Rektman et al. [23] showed that three LCNEC subtypes could be considered: (1) “NSCLC-like” defined by *TP53* and *KRAS*/*STK11*/*KEAP1*, (2) “SCLC-like” based on concurrent *TP53* and *RB1* mutations, and (3) “Carcinoid-like” with *MEN1* mutations. Similarly, George et al. [12] showed LCNECs after molecular analysis could be divided in: Type I LCNECs based on *TP53* and *STK11*/*KEAP1* and Type II LCNECs defined by *TP53*/*RB1* concurrent alterations. These evidences could be used to drive therapeutic approach in particular as underlined by Derks et al. [27].

Moreover, transcriptional studies by Simbolo et al. [24] extended these results proposing a potential grey zone between ACs and LCNECs.

Considering the huge impulse given by molecular studies in improving the proper LNENs definition, in order to identify the best therapeutic approach and the potential role of novel therapies, we performed this Review, supported by in silico analysis, with the aim of tidying up on the mutational status of each WHO 2015 class and to show if the recent molecular novelties could help in better address LNENs treatment.

## 2. Materials and Methods

### 2.1. Study Design

In silico study of LNENs *NGS* datasets of original papers and cBioPortal for Cancer Genomics (https://cbioportal.org) generated by whole genome (WGS), whole exome (WES) and target exome analysis (T-NGS) approach. Datasets of original papers were obtained with a systematic search on PubMed electronic database that were published from 2014 to 2019. The introduction of visualization features of the cBioPortal for Cancer Genomics was published previously [28].

### 2.2. Paper and Datasets Selection

We conducted on PubMed an advanced search using the Advanced Search Builder interface and Boolean operators (AND, OR, NOT). From 2014 to 2019, using the string [(“neuroendocrine lung tumors”) OR (“lung neuroendocrine tumors”) OR (“typical carcinoids”) OR (“atypical carcinoids”) OR (“pulmonary neuroendocrine”) OR (“carcinoid of the lung”) OR (“pulmonary carcinoids”) OR (“large cell neuroendocrine carcinoma of the lung”) OR (“large cell neuroendocrine lung cancer”) OR (“pulmonary large cell neuroendocrine carcinoma”) AND (“next generation sequencing”) OR (“sequencing”) OR (“ngs”) OR (“mutational analysis”) OR (“genomic alterations”) OR (“genomic profiling”)] we found 51 papers. Twelve of them had available original NGS data and were selected for the analysis (Table 1) [10,11,12,13,23,24,27,29,30,31,32,33,34]. On cBioPortal we found and selected only one NGS dataset of SCLC [13], since 2014 until 2019, with the string “lung neuroendocrine” and discarding datasets on cell line and/or PDX models. All the selected datasets had called mutations.

### 2.3. Samples Selection

We included in the analysis the samples whose initial diagnosis was re-evaluated by expert pathologists and classified as TCs, ACs, LCNECs and SCLCs, according WHO guidelines.

### 2.4. Genes Selection and Dataset Creation

In order to investigate pivotal genes in each histological class and coping with the heterogeneity of the different sequencing approaches we kept (i) all gene mutations identified by T-NGS; (ii) all gene mutations identified in NETs (due to their low mutational rate); (iii) only frequently and/or significantly genes mutations in NECs (due to their high mutational rate). For these, we considered variant allele frequency (VAF) until 0.05.

To identify a significant panel of genes, we considered “rare mutations” and eliminated from the analysis all genes with an absolute mutation number ≤ 2.

We generated a new dataset in which all the genes of the defined panel were called for each sample in all analyzed datasets.

### 2.5. Mutation Rate

We defined mutational rate of each gene contained in the panel in each histological class multiplying by 100 the number of detected mutations in each class divided by number of histological samples (TCs, ACs, LCNECs and SCLCs, respectively) in which that gene was sequenced.

$$Mutational\;rate\;\left[\%\right]=\frac{detect\;mutations\times 100}{n\;{}^{\circ}samples\;histological\;group}$$

### 2.6. Statistical Analysis

All statistical analyses were performed using the R environment for statistical computing and graphics (R Foundation, Vienna, Austria, Version 3.6.2). Comparisons of mutation rates, somatic coding mutation per case, type and number of mutations among groups were performed by Kruskal–Wallis test for counts and Fisher exact test for categorical variables. All tests were two-sided and *p*-values < 0.05 were considered statistically significant. Molecular Signature Database (MSigDB) online tool was used to highlight common processes, pathways, and underlying biological themes [35]. We overlapped genes with mutation frequency > 1% detected in each class with all MSigDB collections available and selected top 10 gene set with FDR *q*-value below 0.01.

## 3. Results

### 3.1. Number of Samples, Genes and Mutation Rate across WHO Histological Variants

**Figure 1:**
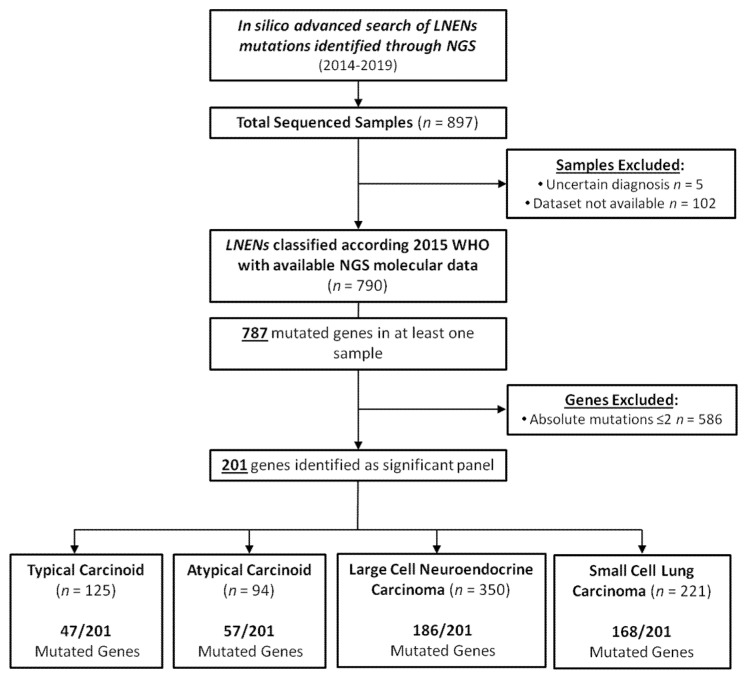
Selection of samples and genes for in Silico molecular analysis. Abbreviations: LNENs, lung neuroendocrine neoplasms; NGS, Next-generation sequencing.

We analyzed 790 samples distributes as follow: 125 TC, 94 AC, 350 LCNEC and 221 SCLC. We found 787 mutated genes in at least one case. We identified a panel of 201 genes after removing rarely mutated genes, i.e., with an absolute mutation number ≤ 2 and calculated the mutation rate of each gene in each histological class (Figure 1).

**Figure 2:**
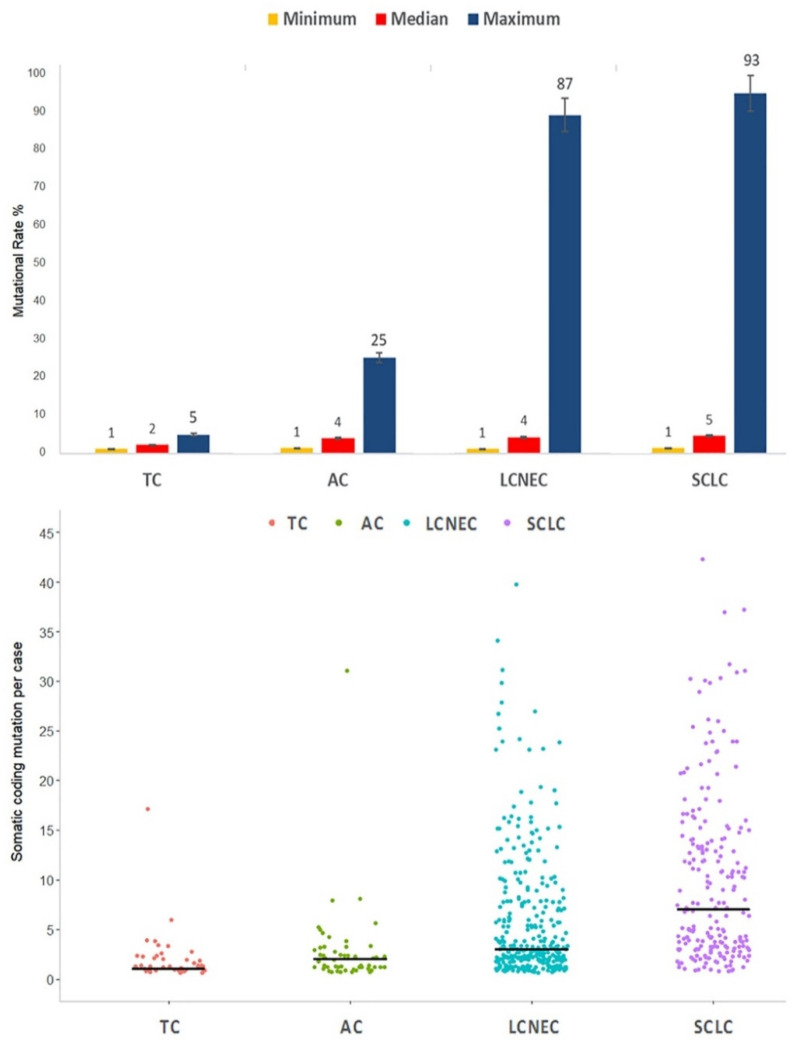
Minimum, median and maximum mutation rate (**above**) and somatic coding mutation per case (**below**) in all four histological variants. Abbreviations: TC, typical carcinoid; AC, atypical carcinoid; LCNEC, large cell neuroendocrine carcinoma; SCLC, small cell lung carcinoma.

The four classes showed both significantly different mutation rate and somatic coding mutation per case. Specifically, TC showed very low mutation rate with maximum range of 4.9% and median of 1 mutation per case; AC highlighted its intermediate-grade with maximum range of 24.7% and 2 median mutations per case; LCNEC and SCLC showed high mutation rate with maximum ranges of 87.1% and 92.8% and a median per case mutation of 3 and 7, respectively (Tables S1–S4; Table 2; Figure 2). Interestingly, TC and AC showed a high maximum range of 17 and 31 somatic coding mutations per case.

Missense mutations are predominant in all histological classes with a little higher frequency in TC and AC than LCNEC and SCLC. On the contrary nonsense mutations are enriched in the latter. Frameshift and splice mutations are more represented in AC and TC, respectively. In SCLCs, we observed also sporadic non-Stop mutations (0.1%). Mutations type showed statistical differences among histological groups (Table 2).

### 3.2. Altered Genes and Pathways in NETs

In both NETs, mutations involved mainly in covalent histone modification and chromatin remodeling process where found. On the contrary, low frequencies were observed in genes related to lung cancer and regulation of cellular processes.

Specifically, TC showed 47 genes with mutation rate at least >1.1% (Table S1). Most mutated genes were EIF1AX (4.84%), ARID1A (4.71%), LRP1B (4.35%) and NF1 (3.53%). Other mutated genes had a mutation frequency <3% and include classical cancer related genes (KRAS, SMAD4, PDGFRB, KIT, APC, ERBB2, AR, MSH3 and LAMA1), assembly and disassembly of chromosomes (ATM, MEN1, FANCD2, KAT6B, RAD51C, PARP1, KMT2C, HECW2, BAP1 and POLE) and signal transmission (ROS1, EPHA3, FLT).

AC showed 57 genes with mutation rate at least >1.1% (Table S2). MEN1 (24.66%) was the most mutated gene following by EIF1AX (16.67%), ARID1A (9.59%) and SMARCA4 (9.59%). Other mutated genes with mutation rate ≥10% were ATP1A2 (18.18%) and SPHKAP (12.82%). Mutations less than 10% comprehend also classical lung cancer related genes (EGFR, PDGFRA, NF1, KRAS, NTRK3, APC, GNAS, KDR, ERBB4 and EPHA5) and regulation of cellular processes (ERBB2, FGFR1, FLT4, RET, KIT, PTEN, PIK3CA, KDR, EPHB1, NOTCH2, TNFAIP3, AR, PTPRT, SMO, PTCH1, DICER1, PTPRZ1, CSMD3 and MDM4).

**Figure 3:**
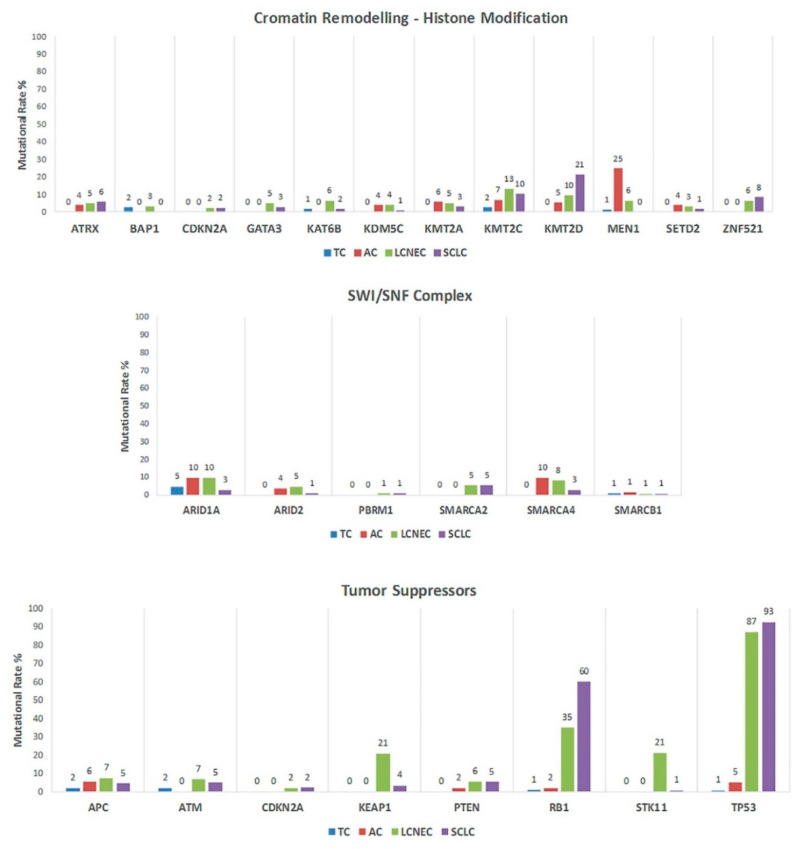
Mutation rate of tumor suppressors and chromatin modifiers genes in all four histological variants. Abbreviations: TC, typical carcinoid; AC, atypical carcinoid; LCNEC, large cell neuroendocrine carcinoma; SCLC, small cell lung carcinoma.

Mutations in tumor suppressor gene TP53 and RB1 are 0.81% and 0.98% in TC and are 5.32% and 2.22% in AC, respectively (Figure 3).

Interestingly EIF1AX mutations were enriched in both NETs and completely absent in LCNEC and SCLC (*p* = 0.0004, Table S5). ARID1A was observed in both NETs and NECs but with significantly higher mutational rate in AC and LCNEC (*p* = 0.02). MEN1 mutations on the other hand was significantly enriched in AC compared to TC (24.66% vs. 1.17%; *p* < 0.0001). A low mutational rate of this gene was also observed in LCNECs (6.33%), while completely absent in SCLC. A higher mutational rate in AC were detected in SMARCA4, AMER1 and RAD51C mutations with statistical differences between the four groups (Table S5). ATP1A2 mutations also showed a higher mutational rate in AC but without statistical significance.

### 3.3. Altered Genes and Pathways in NEC

NECs showed many mutated genes with a high mutation rate compared to NETs. In detail, LCNEC and SCLC showed 186 and 221 mutated genes with mutation rate at least >1.1%, respectively (Tables S3 and S4). The most frequent alterations concern the tumor suppressor genes which have been reported in several types of cancer.

TP53 was the most recurrently mutated gene and significantly enriched in both NECs with mutational rate of 87.14% in LCNEC and 92.76% in SCLC compared to 0.81% of TC and 5.31% of AC (*p* < 0.0001). Mutations in *RB1*, *LRP1B*, *CSMD3*, *SYNE1* and *USH2A* were also significantly enriched in NECs compared to NETs (*p* < 0.0001, Table S5).

*STK11* and *KEAP1* mutations were almost exclusive of LCNECs (19.49% and 19.91%), rare in SCLC (0.76% and 3.50%) and completely absent in both NETs. Contrarily *ZFHX4*, *SPHKAP*, *KMT2D* and *KIAA1211* were statistically enriched in SCLCs compared to the other three classes.

Interestingly, both NECs showed mutations chromatin-remodeling genes and SWItching/Sucrose Non-Fermentable (SWI/SNF) with variable mutation rate (Figure 3). These mutations had usually higher rate in LCNECs than SCLCs. Exception for *KMT2D* which was enriched in SCLC (*p* = 0.0001). In addition, LCNECs showed relevant mutation rate also extracellular matrix genes like *ADAMTS12* (21.67%), *ADAMTS2* (18.33%) and *COL22A1* (10.87%).

## 4. Discussion

LNENs classes according the WHO 2015 [1,2,7], even if further dissected and defined by several studies highlighting their genomic changes and molecular alterations, are not completely able to provide prognostic and therapeutic indications. However, the standard therapeutic approach commonly used in locally advanced and metastatic NETs are completely different than NECs in clinical practice.

**Figure 4:**
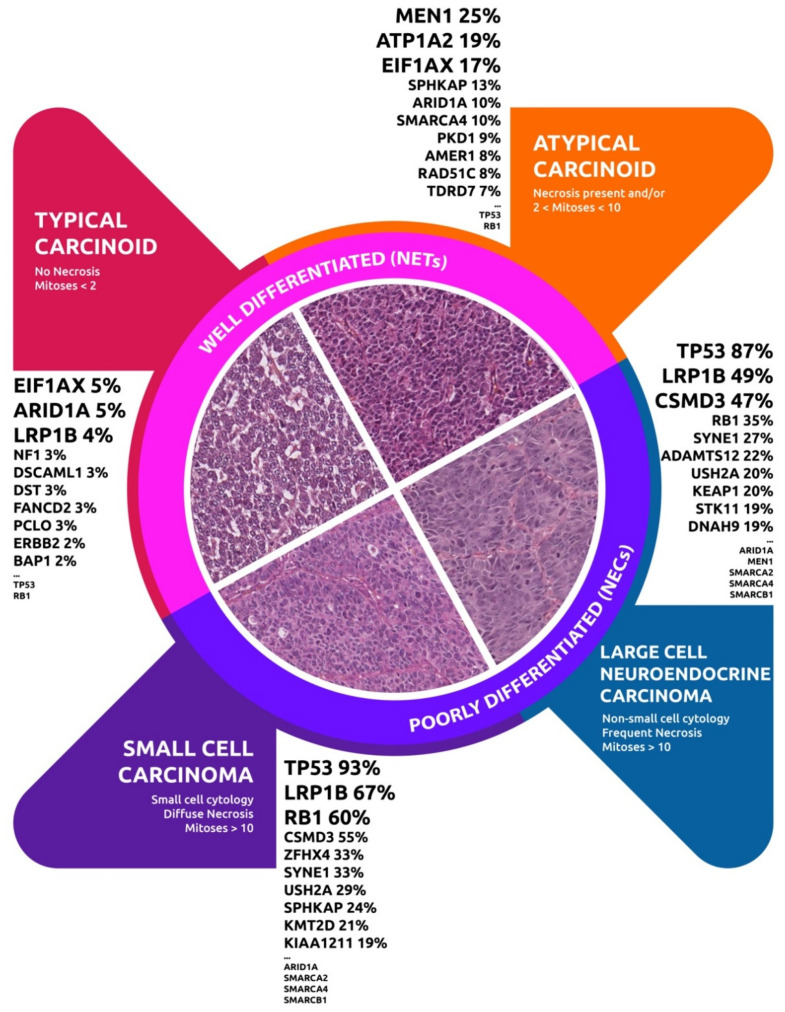
Genomic alterations among the LNENS World Health Organization 2015 groups.

In this review, trough in silico analysis we investigated the available genomic datasets regarding LNENs as at present defined by the World Health Organization (Figure 4).

We showed that NECs compared to NETs are characterized by a considerably higher number of gene alterations and mutational rate. Mutations in *TP53* and *RB1* suppressor gene are present in all classes, but significantly enriched in NECs (*p* < 0.0001). Regarding NETs, the highest significantly enriched mutational rate are of *EIF1AX,* that encodes an essential eukaryotic translation initiation factor, and of genes involved in the chromatin-remodeling and SWI/SNF complex subunit, such as *MEN1* and *ARID1A*. These genes are also involved in NECs (Figure 3). These findings suggest that tumor suppressor *TP53*, *RB1* and chromatin modifiers could be mutated in all four histological variants but with different mutation rate between NECs and NETs; it is expected that these genes play a pivotal role in the pathogenesis of all LNENs. A direct comparison of all histological groups by Simbolo et al. [10] on 148 LNETS highlighted the predominant role of chromatin modifiers in all LNENS, suggesting that their role in NETs could be more relevant, due to their low mutational background [10]. In NECs they can be insignificant due to a high mutational background highly related to smoking, and mutations in different oncogenes and tumor suppressors, such as *TP53* and *RB1* [10,12,13,23,24,31].

Many studies have shown that tumors with higher mutation load respond better to immunotherapy [36,37]. High rate of mutations could result in the formation of neoantigens, which is hypothesized to enhance the anti-tumor immune response [37]. In NETs, both the anti-PD1 agents pembrolizumab and spartalizumab have proven to be safe, but the results are unsatisfactory in terms of activity [38,39]. We observed low somatic coding mutation per case for both NETs, but also higher mutation load (≥6 somatic mutations) for two TCs and four ACs. Interestingly, one TC and one AC showed hypermutated profiles (17 and 31 somatic mutations per case, respectively) that could potentially be highly responsive to the immunotherapy. In SCLC, the use of immunotherapy in combination with chemotherapy has become the new therapeutic first line standard in advanced disease. Nevertheless, immunotherapy alone in SCLC did not provide the groundbreaking results obtained in NSCLC and we have few data regarding the role of immunotherapy in LCNECs [40]. Recently Sherman et al. [41] reported an objective response rate of 33% and a median progression free survival of 4.9 months in a group of 37 consecutive LCNECs treated with immune check-point inhibitors and concluded that the outcomes are comparable with the outcomes reported in advanced NSCLC. Other studies are needed to validate these preliminary results, but for the purpose to select candidate LCNEC patients for immunotherapy, the classification of LCNEC in different genomic subtypes (i.e., “NSCLC-like” characterized by *TP53* and *KRAS*/*STK11*/*KEAP1*, “SCLC-like” with concurrent *TP53* and *RB1*, and “Carcinoid-like” with *MEN1* mutations) could play a very important role [23].

This classification, as well as other similar genomic classification (Karlsson et al. [25], Derks, J.L. et al. [27] and George et al. [12]), may also help clinicians in choosing the best chemotherapy for LCNEC patients. Due to the clinical similarity of LCNEC and SCLC, for years, etoposide/platinum combination has been the gold standard first line approach in advanced LCNECs. However in recent years, several authors are starting to propose that “NSCLC-like” advanced LCNECs should be treated, both in first- and second-line setting, with the chemotherapy regimens used for the treatment of NSCLC [20]. In particular, Derks et al. [27], showed that patients with *RB1* wild-type (WT) LCNEC treated with NSCLC chemotherapy regimens had a significantly longer overall survival, compared to patients treated with SCLC chemotherapy regimens (9.6 vs. 5.8, respectively). Similar results were obtained for patients expressing *RB1* in their tumors [27]. In agreement with our data, *RB1* mutations occurs in 35% of LCNECs as well as typical NSCLC mutations such as *KEAP1* and *STK11* only occur in this tumor variants compared to both NETs and SCLCs (*p* < 0.0001).

In SCLC and “SCLC like” LCNECs the inactivation of *TP53* prevents oncogene-induced senescence and the inactivation of *RB1* leads to increase in cellular proliferation due to loss of cell cycle control [13,42]. This result could be considered as driver for new targeted therapeutic strategies. Due to their lack of functional *RB1* the majority of SCLC models are insensitive to the CDK4/6 inhibitors [43,44]. In contrast, some SCLC models with functional *RB1* are sensitive to the CDK4/6 inhibitors [45]. Based on these data patients with “SCLC like” LCNEC subtype with WT *RB1*, WT *KEAP1*, WT *STK11* and loss of neuroendocrine markers, could benefit from CDK4/6 inhibitors.

As the distinction between SCLCs and LCNECs is nuanced, so there are genetic and transcriptional overlaps between LCNECs and ACs too. Interestingly, *MEN1* is the most frequently mutated gene in ACs (mutated in 24.66%, 18/73 of ACs) and between the two NECs this mutation is occasional in LCNECs (6.3%, 9/142 of LCNECs) and completely absent in SCLCs (*p* < 0.0001). Similarly, Swarts DR et al. [26] and Simbolo et al. [10] reported *MEN1* mutations in 22% (5/23) and 20% (7/35) of ACs versus 14% (1/7) and 4% (1/27) of LCNECs, respectively. Recently, a new paper by Simbolo et al. [24] demonstrated that ACs and LCNECs should be classified in three different subgroups: LCNEC-enriched subgroup whose hallmark is *RB1* inactivation, AC-enriched subgroup in which *MEN1* inactivation plays a major role and a mixed group with intermediate molecular features. ACs and LCNECs are recognized to show borderline characteristics. Increasingly, papers have reported that subgroups of LCNECs may show low proliferation activity given by a low mitotic number or Ki−67 index and share morphological characteristics of carcinoids that are in fact in a gray area for classification [46,47]. Moreover, recent work carried out by Alcala et al. [48] showed that the separation between NETs and LCNECs might be more subtle than initially thought and identified a subgroup of ACs, named supra-carcinoids, that has carcinoid-like morphological pattern but with molecular characteristics similar to LCNECs. On the other side, our data also demonstrated that TCs and ACs can show hypermutated profiles might overlap with supra-carcinoids above mentioned. This leads us to conclude that the best therapeutic strategy for LNENs should not be only based on the current histological classification but also should be tailored on the genetic and transcriptional characteristics of the single tumor. Given these premises the use of platinum-based or temozolomide-based chemotherapy could perhaps be more indicated in the ACs genetically more like LCNECs while SSAs, PRRT and everolimus may be used as first step, in the ACs genetically more like TCs.

In this context, chromatin-modifying genes could also play a major role in LNENs in the near future, mainly in NETs. In particular, *ARID1A* gene is a member of the SWI/SNF complex involved in chromatin remodeling. Alterations in the *ARID1A* gene lead to its inactivation with a consequent protein function loss. These alterations were reported in different cancer types [49,50]. We observed *ARID1A* mutations in all four histological variants but significantly enriched in ACs and LCNECs (*p* = 0.02). The literature showed that *ARID1A* alterations may sensitize tumors to agents targeting the ATR protein, EZH2 or the PI3K pathway, but we have only preliminary results [51,52,53]. *ARID1A* alterations also compromise the mismatch repair interacting with MSH2 protein and ARID1A-deficient ovarian cancer cell line in syngeneic mice exhibited an increased number of tumor-infiltrating lymphocytes, a higher tumor mutation load and elevated PD-L1 levels [49]. Thus, *ARID1A* mutated tumors especially with elevated PD-L1 levels, microsatellite instability-high and high tumor mutational burden could be good candidate to anti-PD-1/PD-L1 immunotherapy as demonstrated by a very recent report [54].

## 5. Conclusions

In summary, in this review, through in silico analysis, we show that, due to the rarity and molecular heterogeneity, the addition of a molecular layer to the current WHO classification could have a very positive impact on the therapeutic approach to LNENs. In addition to what has been widely reported in the literature, that NETs and NECs are characterized by a low and high mutational load, respectively, this data confirmed that mutations in chromatin modifying genes and tumor suppressors *TP53*-*RB1* are represented in all classes with different mutation rate, suggesting a pivotal role in the pathogenesis of all LNENs and possible future therapeutic implications. In future, the integration of CNAs and RNAseq analysis with big standardized datasets and/or specific histological variants will allow us to better identify the genomic regions involved in LNEN carcinogenesis, in order to develop new knowledge on possible therapeutic implications in these four histological variants. In conclusion, a new classification of LNENs that takes into account the genomic and transcriptional data emerging from the literature is strongly suggested and could have a very positive impact on the therapeutic approach to LNENs.

## Figures and Tables

**Table 1:** Studies with available original next generation sequencing data selected for the analysis.

Year	Author	Sequenced Samples (*n*)	Sample Histotype (*n*)	WGS (*n*)	WES (*n*)	t-NGS (*n*)	Genes Analyzed	Sequencing	Selected Samples (*n*)
2014	Fernandez-Cuesta et al. [11]	44	TC (34)	TC (24)	TC (10)	0	All	WGS and WES	34 TC 5 AC
			AC (5)	AC (1)	AC (4)				
			CA NAS (5)	CA NAS (4)	CA NAS (1)				
2015	Armengol et al. [29]	25	TC (21)	0	0	TC (21)	22 (t-NGS)	Ion AmpliSeq Colon and Lung Cancer Research Panel v2 (Thermofisher)	TC (21)
			AC (4)			AC (4)			AC (4)
2015	Karlsson et al. [30]	32	LCNEC (32)	0	0	LCNEC (32)	26 (t-NGS)	Illumina TruSight Tumor 26-gene next-generation sequencing (NGS) panel (Illumina). LCNEC cases were screened for retinoblastoma 1 gene (*RB1*) mutations by using a custom-designed bidirectional NGS panel (Illumina).	LCNEC (32)
2015	Vollbrecht et al. [31]	70	TC (17)	0	0	TC (17)	48 (t-NGS)	TruSeq Amplicon–Cancer Panel (Illumina, San Diego, CA, USA)	TC (17)
			AC (17)			AC (17)			AC (17)
			LCNEC (19)			LCNEC (19)			LCNEC (19)
			SCLC (17)			SCLC (17)			SCLC (17)
2015	George et al. [13]	110	SCLC (110)	SCLC (110)	0	0	All	WGS	110 SCLC
2016	Rekhtman et al. [23]	45	LCNEC (45)	0	0	LCNEC (45)	241 (t-NGS)	Memorial Sloan Kettering-Integrated Mutation Profiling of Actionable Cancer Targets (MSK-IMPACT) platform	45 LCNEC
2017	Miyoshi et al. [32]	168	LCNEC (78)	0	0	LCNEC (78)	244 (t-NGS)	Custom target-capturing panel (SureSelect XT custom 0.5–2.9 Mb, Agilent Technologies) containing all the coding exons of 244 genes	LCNEC (78)
			SCLC (90)			SCLC (90)			SCLC (90)
2017	Simbolo et al. [10]	148	TC (53)	0	TC (10)	TC (43)	All (WES)	WES and Ion AmpliSeq Comprehensive Cancer Panel (ThermoFisher)	TC (23)
			AC (35)		AC (4)	AC (31)	418 (HCTS) *		AC (14)
			LCNEC (27)		LCNEC (3)	LCNEC (24)	88 (t-NGS)		LCNEC (5)
			SCLC (33)		SCLC (3)	SCLC (30)			SCLC (4)
2018	Derks et al. [27]	79	LCNEC (79)	0	0	LCNEC (79)	4 (t-NGS)	Qiagen GeneRead DNAseq Custom V2 Builder (*TP53*, *RB1*, *STK11*, and *KEAP1*)	LCNEC (79)
2018	Asiedu et al. [33]	20	TC (14)	TC (3)	TC (14)	0	All	WGS and WES	TC (14)
			AC (6)	AC (2)	AC (6)				AC (6)
2018	George et al. [12]	60	LCNEC (60)	LCNEC (11)	LCNEC (55)	0	All	WGS and WES	60 LCNEC
2019	Simbolo et al. [24]	67	AC (35)	0	0	AC (35)	409 (HCTS) *	Ampliseq Transcriptome Human Gene Expression Kit (ThermoFisher); Ampliseq Comprehensive Cancer Panel (ThermoFisher)	AC (14) HTCS AC (21) t-NGS LCNEC (14) HTCS LCNEC (18) t-NGS
			LCNEC (32)			LCNEC (32)	13 (t-NGS)		
2019	Saurabh V. Laddha et al. [34]	29	TC (16) AC (13)	0	0	TC (16) AC (13)	354 (t-NGS)	MSK-IMPACT	TC (16) AC (13)

* = high-coverage targeted sequencing.

**Table 2:** Characteristics of mutated genes among WHO histological variants.

Features	All	TCs	ACs	LCNECs	SCLCs	*p*-Value *
Total Mutated Genes ^†^	201	47	57	186	168	-
Mutational Rate %						
Median [range]	4.00 (1.14–92.76)	2.17 (1.18–4.84)	3.85 (1.37–24.66)	4.22 (1.14–87.14)	4.55 (1.36–92.76)	<0.0001
Somatic coding mutation per case						
Median [range]	3 (1–42)	1 (1–17)	2 (1–31)	3 (1–40)	7 (1–42)	<0.0001
Type of Mutations						
Missense	2958 (69.8)	66 (75.9)	107 (73.8)	1299 (68.8)	1486 (70.1)	
Nonsense	553 (13.05)	6 (6.9)	10 (6.9)	284 (15.1)	253 (11.9)	
Frameshift	503 (11.9)	7 (8)	24 (16.5)	204 (10.8)	268 (12.7)	
Splice	222 (5.2)	8 (9.2)	4 (2.8)	100 (5.3)	110 (5.2)	
Non-Stop	2 (0.05)	0 (0.0)	0 (0.0)	0 (0.0)	2 (0.1)	0.004

Abbreviations: TCs, typical carcinoids; ACs, atypical carcinoids; LCNECs, large cell neuroendocrine carcinomas; SCLCs, small cell lung carcinomas. Note: * *p*-value based on Fisher exact test for categorical variables or on the Kruskal–Wallis test for counts; ^†^ Only genes with mutation numbers > 2.

## Supplementary Materials

The following are available online at https://www.mdpi.com/2072-6694/12/10/2753/s1, Table S1: Genes frequently mutated in typical carcinoids (TCs), Table S2: Genes frequently mutated in atypical carcinoids (ACs), Table S3: Genes frequently mutated in large cell neuroendocrine carcinomas (LCNECs); Table S4: Genes frequently mutated in small cell lung carcinomas (SCLCs); Table S5: Top 10 recurrently mutated genes among WHO histotypes.
