# Supplementary material for: Beyond Traditional Morphological Characterization of Lung Neuroendocrine Neoplasms: In Silico Study of Next-Generation Sequencing Mutations Analysis across the Four World Health Organization Defined Groups
Source: Cancers (Basel). 2020 Sep 24;12(10):2753. doi: 10.3390/cancers12102753 (PMC7600757; doi:10.3390/cancers12102753)
Supplement: Supplementary file 1 [file cancers-12-02753-s001.pdf]

Beyond Traditional Morphological Characterization of Lung Neuroendocrine Neoplasms: In Silico Study of Next-Generation Sequencing Mutations Analysis across the Four World Health Organization Defined Groups

Giovanni Centonze, Davide Biganzoli, Natalie Prinzi, Sara Pusceddu, Alessandro Mangogna, Elena Tamborini, Federica Perrone, Adele Busico, Vincenzo Lagano, Laura Cattaneo, Gabriella Sozzi, Luca Roz, Elia Biganzoli and Massimo Milione

Table S1. Genes Frequently mutated in Typical Carcinoids (TCs).

Mutation Rate %	Original	Entrez	Gene	Gene
4.84	EIF1AX	1964	EIF1AX	eukaryotic translation initiation factor 1A X-linked [Source: HGNC Symbol; Acc: HGNC: 3250]
4.71	ARID1A	8289	ARID1A	AT-rich interaction domain 1A [Source: HGNC Symbol;Acc: HGNC: 11110]
4.35	LRP1B	53353	LRP1B	LDL receptor related protein 1B [Source: HGNC Symbol; Acc: HGNC: 6693]
3.53	NF1	4763	NF1	neurofibromin 1 [Source: HGNC Symbol;Acc: HGNC: 7765]
2.90	DSCAML1	57453	DSCAML1	DS cell adhesion molecule like 1 [Source: HGNC Symbol; Acc: HGNC: 14656]
2.90	DST	667	DST	dystonin [Source: HGNC Symbol;Acc: HGNC: 1090]
2.90	FANCD2	2177	FANCD2	FA complementation group D2 [Source: HGNC Symbol; Acc: HGNC: 3585]
2.90	PCLO	27445	PCLO	piccolo presynaptic cytomatrix protein [Source: HGNC Symbol; Acc: HGNC: 13406]
2.44	ERBB2	2064	ERBB2	erb-b2 receptor tyrosine kinase 2 [Source: HGNC Symbol; Acc: HGNC: 3430]
2.35	BAP1	8314	BAP1	BRCA1 associated protein 1 [Source: HGNC Symbol; Acc: HGNC: 950]
2.35	CIC	23152	CIC	capicua transcriptional repressor [Source: HGNC Symbol; Acc: HGNC: 14214]
2.35	EPHA3	2042	EPHA3	EPH receptor A3 [Source: HGNC Symbol; Acc: HGNC: 3387]
2.35	MLL3	58508	KMT2C	lysine methyltransferase 2C [Source: HGNC Symbol; Acc: HGNC: 13726]
2.35	PDGFRB	5159	PDGFRB	platelet derived growth factor receptor beta [Source: HGNC Symbol; Acc: HGNC: 8804]
2.35	ROS1	6098	ROS1	ROS proto-oncogene 1, receptor tyrosine kinase [Source: HGNC Symbol; Acc: HGNC: 10261]
2.17	APBB1IP	54518	APBB1IP	amyloid beta precursor protein binding family B member 1 interacting protein [Source: HGNC Symbol; Acc: HGNC: 17379]
2.17	ATP1A2	477	ATP1A2	ATPase Na+/K+ transporting subunit alpha 2 [Source: HGNC Symbol; Acc: HGNC: 800]
2.17	CNTNAP2	26047	CNTNAP2	contactin associated protein like 2 [Source: HGNC Symbol; Acc: HGNC: 13830]
2.17	HECW2	57520	HECW2	HECT, C2 and WW domain containing E3 ubiquitin protein ligase 2 [Source: HGNC Symbol;Acc: HGNC: 29853]
2.17	LAMA1	284217	LAMA1	laminin subunit alpha 1 [Source: HGNC Symbol; Acc: HGNC: 6481]
2.17	MDN1	23195	MDN1	midasin AAA ATPase 1 [Source: HGNC Symbol; Acc: HGNC: 18302]
2.17	MSH3	4437	MSH3	mutS homolog 3 [Source: HGNC Symbol; Acc: HGNC: 7326]
2.17	NCOR2	9612	NCOR2	nuclear receptor corepressor 2 [Source: HGNC Symbol; Acc: HGNC: 7673]
2.17	SALL1	6299	SALL1	spalt like transcription factor 1 [Source: HGNC Symbol; Acc: HGNC: 10524]
1.96	APC	324	APC	APC regulator of WNT signaling pathway [Source: HGNC Symbol; Acc: HGNC: 583]

1.96	ATM	472	ATM	ATM serine/threonine kinase [Source: HGNC Symbol; Acc: HGNC: 795]
1.96	IDH1	3417	IDH1	isocitrate dehydrogenase (NADP(+)) 1 [Source: HGNC Symbol; Acc: HGNC: 5382]
1.63	KRAS	3845	KRAS	KRAS proto-oncogene, GTPase [Source: HGNC Symbol; Acc: HGNC: 6407]
1.63	SMAD4	4089	SMAD4	SMAD family member 4 [Source: HGNC Symbol; Acc: HGNC: 6770]
1.61	FAT1	2195	FAT1	FAT atypical cadherin 1 [Source: HGNC Symbol; Acc: HGNC: 3595]
1.61	NOTCH3	4854	NOTCH3	notch receptor 3 [Source: HGNC Symbol; Acc: HGNC: 7883]
1.61	PARP1	142	PARP1	poly(ADP-ribose) polymerase 1 [Source: HGNC Symbol; Acc: HGNC: 270]
1.61	POLE	5426	POLE	DNA polymerase epsilon, catalytic subunit [Source: HGNC Symbol; Acc: HGNC: 9177]
1.61	RAD51C	5889	RAD51C	RAD51 paralog C [Source: HGNC Symbol; Acc: HGNC: 9820]
1.61	SF3B1	23451	SF3B1	splicing factor 3b subunit 1 [Source: HGNC Symbol; Acc: HGNC: 10768]
1.45	CSMD3	114788	CSMD3	CUB and Sushi multiple domains 3 [Source: HGNC Symbol; Acc: HGNC: 19291]
1.45	KAT6B	23522	KAT6B	lysine acetyltransferase 6B [Source: HGNC Symbol; Acc: HGNC: 17582]
1.45	NCAM2	4685	NCAM2	neural cell adhesion molecule 2 [Source: HGNC Symbol; Acc: HGNC: 7657]
1.45	PDE4DIP	9659	PDE4DIP	phosphodiesterase 4D interacting protein [Source: HGNC Symbol; Acc: HGNC: 15580]
1.45	SPHKAP	80309	SPHKAP	SPHK1 interactor, AKAP domain containing [Source: HGNC Symbol; Acc: HGNC: 30619]
1.45	SYNE1	23345	SYNE1	spectrin repeat containing nuclear envelope protein 1 [Source: HGNC Symbol; Acc: HGNC: 17089]
1.45	TDRD7	23424	TDRD7	tudor domain containing 7 [Source: HGNC Symbol; Acc: HGNC: 30831]
1.27	KIT	3815	KIT	KIT proto-oncogene, receptor tyrosine kinase [Source: HGNC Symbol; Acc: HGNC: 6342]
1.18	AR	367	AR	androgen receptor [Source: HGNC Symbol; Acc: HGNC: 644]
1.18	FLT1	2321	FLT1	fms related tyrosine kinase 1 [Source: HGNC Symbol; Acc: HGNC: 3763]
1.18	MEN1	4221	MEN1	menin 1 [Source: HGNC Symbol; Acc: HGNC: 7010]
1.18	NOTCH2	4853	NOTCH2	notch receptor 2 [Source: HGNC Symbol; Acc: HGNC: 7882]

Table S2. Genes Frequently mutated in Atypical Carcinoids (ACs).

Mutation Rate %	Original	Entrez	Gene	Gene
24.66	MEN1	4221	MEN1	menin 1 [Source: HGNC Symbol; Acc: HGNC: 7010]
18.18	ATP1A2	477	ATP1A2	ATPase Na+/K+ transporting subunit alpha 2 [Source: HGNC Symbol; Acc: HGNC: 800]
16.67	EIF1AX	1964	EIF1AX	eukaryotic translation initiation factor 1A X-linked [Source: HGNC Symbol; Acc: HGNC: 3250]
12.82	SPHKAP	80309	SPHKAP	SPHK1 interactor, AKAP domain containing [Source: HGNC Symbol; Acc: HGNC: 30619]
9.59	ARID1A	8289	ARID1A	AT-rich interaction domain 1A [Source: HGNC Symbol; Acc: HGNC: 11110]
9.59	SMARCA4	6597	SMARCA4	SWI/SNF related, matrix associated, actin dependent regulator of chromatin, subfamily a, member 4 [Source: HGNC Symbol; Acc: HGNC: 11100]
9.09	PKD1	5310	PKD1	polycystin 1, transient receptor potential channel interacting [Source: HGNC Symbol; Acc: HGNC: 9008]
8.33	AMER1	139285	AMER1	APC membrane recruitment protein 1 [Source: HGNC Symbol; Acc: HGNC: 26837]
8.33	RAD51C	5889	RAD51C	RAD51 paralog C [Source: HGNC Symbol; Acc: HGNC: 9820]
7.69	TDRD7	23424	TDRD7	tudor domain containing 7 [Source: HGNC Symbol; Acc: HGNC: 30831]
6.85	MLL3	58508	KMT2C	lysine methyltransferase 2C [Source: HGNC Symbol; Acc: HGNC: 13726]
6.85	NOTCH2	4853	NOTCH2	notch receptor 2 [Source: HGNC Symbol; Acc: HGNC: 7882]

5.80	APC	324	APC	APC regulator of WNT signaling pathway [Source: HGNC Symbol; Acc: HGNC: 583]
5.80	PDGFRA	5156	PDGFRA	platelet derived growth factor receptor alpha [Source: HGNC Symbol; Acc: HGNC: 8803]
5.77	MLL	4297	KMT2A	lysine methyltransferase 2A [Source: HGNC Symbol; Acc: HGNC: 7132]
5.48	MLL2	8085	KMT2D	lysine methyltransferase 2D [Source: HGNC Symbol; Acc: HGNC: 7133]
5.32	TP53	7157	TP53	tumor protein p53 [Source: HGNC Symbol; Acc: HGNC: 11998]
5.26	SF3B1	23451	SF3B1	splicing factor 3b subunit 1 [Source: HGNC Symbol; Acc: HGNC: 10768]
5.13	CSMD3	114788	CSMD3	CUB and Sushi multiple domains 3 [Source: HGNC Symbol; Acc: HGNC: 19291]
5.13	DSCAML1	57453	DSCAML1	DS cell adhesion molecule like 1 [Source: HGNC Symbol; Acc: HGNC: 14656]
5.13	NCAM2	4685	NCAM2	neural cell adhesion molecule 2 [Source: HGNC Symbol; Acc: HGNC: 7657]
5.13	PCLO	27445	PCLO	piccolo presynaptic cytomatrix protein [Source: HGNC Symbol; Acc: HGNC: 13406]
5.13	PTPRZ1	5803	PTPRZ1	protein tyrosine phosphatase receptor type Z1 [Source: HGNC Symbol; Acc: HGNC: 9685]
4.35	HNF1A	6927	HNF1A	HNF1 homeobox A [Source: HGNC Symbol; Acc: HGNC: 11621]
4.35	KDR	3791	KDR	kinase insert domain receptor [Source: HGNC Symbol; Acc: HGNC: 6307]
4.17	EPHA5	2044	EPHA5	EPH receptor A5 [Source: HGNC Symbol; Acc: HGNC: 3389]
4.17	GRIN2A	2903	GRIN2A	glutamate ionotropic receptor NMDA type subunit 2A [Source: HGNC Symbol; Acc: HGNC: 4585]
3.85	ARID2	196528	ARID2	AT-rich interaction domain 2 [Source: HGNC Symbol; Acc: HGNC: 18037]
3.85	ATRX	546	ATRX	ATRX chromatin remodeler [Source: HGNC Symbol; Acc: HGNC: 886]
3.85	KDM5C	8242	KDM5C	lysine demethylase 5C [Source: HGNC Symbol; Acc: HGNC: 11114]
3.85	PTPRT	11122	PTPRT	protein tyrosine phosphatase receptor type T [Source: HGNC Symbol; Acc: HGNC: 9682]
3.85	SETD2	29072	SETD2	SET domain containing 2, histone lysine methyltransferase [Source: HGNC Symbol; Acc: HGNC: 18420]
3.64	KIT	3815	KIT	KIT proto-oncogene, receptor tyrosine kinase [Source: HGNC Symbol; Acc: HGNC: 6342]
2.74	EGFR	1956	EGFR	epidermal growth factor receptor [Source: HGNC Symbol; Acc: HGNC: 3236]
2.74	ERBB2	2064	ERBB2	erb-b2 receptor tyrosine kinase 2 [Source: HGNC Symbol; Acc: HGNC: 3430]
2.74	ERBB4	2066	ERBB4	erb-b2 receptor tyrosine kinase 4 [Source: HGNC Symbol; Acc: HGNC: 3432]
2.74	FGFR1	2260	FGFR1	fibroblast growth factor receptor 1 [Source: HGNC Symbol; Acc: HGNC: 3688]
2.74	MET	4233	MET	MET proto-oncogene, receptor tyrosine kinase [Source: HGNC Symbol; Acc: HGNC: 7029]
2.56	NUP98	4928	NUP98	nucleoporin 98 [Source: HGNC Symbol; Acc: HGNC: 8068]
2.56	RNF213	57674	RNF213	ring finger protein 213 [Source: HGNC Symbol; Acc: HGNC: 14539]
2.22	RB1	5925	RB1	RB transcriptional corepressor 1 [Source: HGNC Symbol; Acc: HGNC: 9884]
2.13	PIK3CA	5290	PIK3CA	phosphatidylinositol-4,5-bisphosphate 3-kinase catalytic subunit alpha [Source: HGNC Symbol; Acc: HGNC: 8975]
2.13	PTEN	5728	PTEN	phosphatase and tensin homolog [Source: HGNC Symbol; Acc: HGNC: 9588]
1.92	AR	367	AR	androgen receptor [Source: HGNC Symbol; Acc: HGNC: 644]
1.92	DICER1	23405	DICER1	dicer 1, ribonuclease III [Source: HGNC Symbol; Acc: HGNC: 17098]
1.92	EPHB1	2047	EPHB1	EPH receptor B1 [Source: HGNC Symbol; Acc: HGNC: 3392]
1.92	FLT4	2324	FLT4	fms related tyrosine kinase 4 [Source: HGNC Symbol; Acc: HGNC: 3767]
1.92	MDM4	4194	MDM4	MDM4 regulator of p53 [Source: HGNC Symbol; Acc: HGNC: 6974]
1.92	NF1	4763	NF1	neurofibromin 1 [Source: HGNC Symbol; Acc: HGNC: 7765]

1.92	NTRK3	4916	NTRK3	neurotrophic receptor tyrosine kinase 3 [Source: HGNC Symbol; Acc: HGNC: 8033]
1.92	PTCH1	5727	PTCH1	patched 1 [Source: HGNC Symbol; Acc: HGNC: 9585]
1.92	TNFAIP3	7128	TNFAIP3	TNF alpha induced protein 3 [Source: HGNC Symbol; Acc: HGNC: 11896]
1.45	GNAS	2778	GNAS	GNAS complex locus [Source: HGNC Symbol; Acc: HGNC: 4392]
1.45	RET	5979	RET	ret proto-oncogene [Source: HGNC Symbol; Acc: HGNC: 9967]
1.45	SMARCB1	6598	SMARCB1	SWI/SNF related, matrix associated, actin dependent regulator of chromatin, subfamily b, member 1 [Source: HGNC Symbol; Acc: HGNC: 11103]
1.45	SMO	6608	SMO	smoothened, frizzled class receptor [Source: HGNC Symbol; Acc: HGNC: 11119]
1.37	KRAS	3845	KRAS	KRAS proto-oncogene, GTPase [Source: HGNC Symbol; Acc: HGNC: 6407]

Table S3. Genes Frequently mutated in Large Cell Neuroendocrine Carcinomas (LCNECs).

Mutation Rate %	Original	Entrez	Gene	Gene
87.14	TP53	7157	TP53	tumor protein p53 [Source: HGNC Symbol; Acc: HGNC: 11998]
49.37	LRP1B	53353	LRP1B	LDL receptor related protein 1B [Source: HGNC Symbol; Acc: HGNC: 6693]
46.84	CSMD3	114788	CSMD3	CUB and Sushi multiple domains 3 [Source: HGNC Symbol; Acc: HGNC: 19291]
35.22	RB1	5925	RB1	RB transcriptional corepressor 1 [Source: HGNC Symbol; Acc: HGNC: 9884]
26.58	SYNE1	23345	SYNE1	spectrin repeat containing nuclear envelope protein 1 [Source: HGNC Symbol; Acc: HGNC: 17089]
21.67	ADAMTS12	81792	ADAMTS12	ADAM metallopeptidase with thrombospondin type 1 motif 12 [Source: HGNC Symbol; Acc: HGNC: 14605]
20.29	USH2A	7399	USH2A	usherin [Source: HGNC Symbol; Acc: HGNC: 12601]
19.91	KEAP1	9817	KEAP1	kelch like ECH associated protein 1 [Source: HGNC Symbol; Acc: HGNC: 23177]
19.49	STK11	6794	STK11	serine/threonine kinase 11 [Source: HGNC Symbol; Acc: HGNC: 11389]
18.84	DNAH9	1770	DNAH9	dynein axonemal heavy chain 9 [Source: HGNC Symbol; Acc: HGNC: 2953]
18.33	ADAMTS2	9509	ADAMTS2	ADAM metallopeptidase with thrombospondin type 1 motif 2 [Source: HGNC Symbol; Acc: HGNC: 218]
15.32	PTPRT	11122	PTPRT	protein tyrosine phosphatase receptor type T [Source: HGNC Symbol; Acc: HGNC: 9682]
14.52	PTPRD	5789	PTPRD	protein tyrosine phosphatase receptor type D [Source: HGNC Symbol; Acc: HGNC: 9668]
13.18	MLL3	58508	KMT2C	lysine methyltransferase 2C [Source: HGNC Symbol; Acc: HGNC: 13726]
12.38	GRIN2A	2903	GRIN2A	glutamate ionotropic receptor NMDA type subunit 2A [Source: HGNC Symbol; Acc: HGNC: 4585]
11.67	GAS7	8522	AC005747.1	growth arrest specific 7 [Source: NCBI gene; Acc: 8522]
11.67	NTM	50863	NTM	neurotrimin [Source: HGNC Symbol; Acc: HGNC: 17941]
10.93	FAT1	2195	FAT1	FAT atypical cadherin 1 [Source: HGNC Symbol; Acc: HGNC: 3595]
10.87	COL11A1	1301	COL11A1	collagen type XI alpha 1 chain [Source: HGNC Symbol; Acc: HGNC: 2186]
10.87	COL22A1	169044	COL22A1	collagen type XXII alpha 1 chain [Source: HGNC Symbol; Acc: HGNC: 22989]
10.87	ZFHX4	79776	ZFHX4	zinc finger homeobox 4 [Source: HGNC Symbol; Acc: HGNC: 30939]
10.48	EPHA3	2042	EPHA3	EPH receptor A3 [Source: HGNC Symbol; Acc: HGNC: 3387]
10.48	EPHA5	2044	EPHA5	EPH receptor A5 [Source: HGNC Symbol; Acc: HGNC: 3389]
10.14	LAMA2	3908	LAMA2	laminin subunit alpha 2 [Source: HGNC Symbol; Acc: HGNC: 6482]
10.13	DST	667	DST	dystonin [Source: HGNC Symbol; Acc: HGNC: 1090]
10.00	BSN	8927	BSN	bassoon presynaptic cytomatrix protein [Source: HGNC Symbol; Acc: HGNC: 1117]

10.00	FAP	2191	FAP	fibroblast activation protein alpha [Source: HGNC Symbol; Acc: HGNC: 3590]
9.95	NOTCH1	4851	NOTCH1	notch receptor 1 [Source: HGNC Symbol; Acc: HGNC: 7881]
9.55	ARID1A	8289	ARID1A	AT-rich interaction domain 1A [Source: HGNC Symbol; Acc: HGNC: 11110]
9.55	MLL2	8085	KMT2D	lysine methyltransferase 2D [Source: HGNC Symbol; Acc: HGNC: 7133]
8.92	PDE4DIP	9659	PDE4DIP	phosphodiesterase 4D interacting protein [Source: HGNC Symbol; Acc: HGNC: 15580]
8.91	NF1	4763	NF1	neurofibromin 1 [Source: HGNC Symbol; Acc: HGNC: 7765]
8.70	CNTNAP2	26047	CNTNAP2	contactin associated protein like 2 [Source: HGNC Symbol; Acc: HGNC: 13830]
8.57	PAK7	57144	PAK5	p21 (RAC1) activated kinase 5 [Source: HGNC Symbol; Acc: HGNC: 15916]
8.57	POLE	5426	POLE	DNA polymerase epsilon, catalytic subunit [Source: HGNC Symbol; Acc: HGNC: 9177]
8.33	ALMS1	7840	ALMS1	ALMS1 centrosome and basal body associated protein [Source: HGNC Symbol; Acc: HGNC: 428]
8.33	COL5A3	50509	COL5A3	collagen type V alpha 3 chain [Source: HGNC Symbol; Acc: HGNC: 14864]
8.18	SMARCA4	6597	SMARCA4	SWI/SNF related, matrix associated, actin dependent regulator of chromatin, subfamily a, member 4 [Source: HGNC Symbol; Acc: HGNC: 11100]
8.06	NOTCH4	4855	NOTCH4	notch receptor 4 [Source: HGNC Symbol; Acc: HGNC: 7884]
7.97	LRFN5	145581	LRFN5	leucine rich repeat and fibronectin type III domain containing 5 [Source: HGNC Symbol; Acc: HGNC: 20360]
7.97	STAB2	55576	STAB2	stabilin 2 [Source: HGNC Symbol; Acc: HGNC: 18629]
7.91	KRAS	3845	KRAS	KRAS proto-oncogene, GTPase [Source: HGNC Symbol; Acc: HGNC: 6407]
7.69	ERBB4	2066	ERBB4	erb-b2 receptor tyrosine kinase 4 [Source: HGNC Symbol; Acc: HGNC: 3432]
7.62	STAG2	10735	STAG2	stromal antigen 2 [Source: HGNC Symbol; Acc: HGNC: 11355]
7.59	PTPRZ1	5803	PTPRZ1	protein tyrosine phosphatase receptor type Z1 [Source: HGNC Symbol; Acc: HGNC: 9685]
7.43	APC	324	APC	APC regulator of WNT signaling pathway [Source: HGNC Symbol; Acc: HGNC: 583]
7.43	NTRK3	4916	NTRK3	neurotrophic receptor tyrosine kinase 3 [Source: HGNC Symbol; Acc: HGNC: 8033]
6.79	ATM	472	ATM	ATM serine/threonine kinase [Source: HGNC Symbol; Acc: HGNC: 795]
6.67	FMN2	56776	FMN2	formin 2 [Source: HGNC Symbol; Acc: HGNC: 14074]
6.67	IRS1	3667	IRS1	insulin receptor substrate 1 [Source: HGNC Symbol; Acc: HGNC: 6125]
6.67	KIAA1324L	222223	KIAA1324L	KIAA1324 like [Source: HGNC Symbol; Acc: HGNC: 21945]
6.67	PKD1	5310	PKD1	polycystin 1, transient receptor potential channel interacting [Source: HGNC Symbol; Acc: HGNC: 9008]
6.56	BRCA2	675	BRCA2	BRCA2 DNA repair associated [Source: HGNC Symbol; Acc: HGNC: 1101]
6.52	ASPM	259266	ASPM	abnormal spindle microtubule assembly [Source: HGNC Symbol; Acc: HGNC: 19048]
6.52	LAMA1	284217	LAMA1	laminin subunit alpha 1 [Source: HGNC Symbol; Acc: HGNC: 6481]
6.37	ZNF521	25925	ZNF521	zinc finger protein 521 [Source: HGNC Symbol; Acc: HGNC: 24605]
6.34	MEN1	4221	MEN1	menin 1 [Source: HGNC Symbol; Acc: HGNC: 7010]
6.33	KAT6B	23522	KAT6B	lysine acetyltransferase 6B [Source: HGNC Symbol; Acc: HGNC: 17582]
5.80	SALL1	6299	SALL1	spalt like transcription factor 1 [Source: HGNC Symbol; Acc: HGNC: 10524]
5.65	IL7R	3575	IL7R	interleukin 7 receptor [Source: HGNC Symbol; Acc: HGNC: 6024]
5.54	PTEN	5728	PTEN	phosphatase and tensin homolog [Source: HGNC Symbol; Acc: HGNC: 9588]
5.46	NOTCH3	4854	NOTCH3	notch receptor 3 [Source: HGNC Symbol; Acc: HGNC: 7883]

5.43	SMARCA2	6595	SMARCA2	SWI/SNF related, matrix associated, actin dependent regulator of chromatin, subfamily a, member 2 [Source: HGNC Symbol; Acc: HGNC: 11098]
5.10	PCLO	27445	PCLO	piccolo presynaptic cytomatrix protein [Source: HGNC Symbol; Acc: HGNC: 13406]
5.07	PRIC285	85441	HELZ2	helicase with zinc finger 2 [Source: HGNC Symbol; Acc: HGNC: 30021]
5.06	DSCAML1	57453	DSCAML1	DS cell adhesion molecule like 1 [Source: HGNC Symbol; Acc: HGNC: 14656]
5.06	SPHKAP	80309	SPHKAP	SPHK1 interactor, AKAP domain containing [Source: HGNC Symbol; Acc: HGNC: 30619]
5.06	THSD7B	80731	THSD7B	thrombospondin type 1 domain containing 7B [Source: HGNC Symbol; Acc: HGNC: 29348]
5.00	HECW2	57520	HECW2	HECT, C2 and WW domain containing E3 ubiquitin protein ligase 2 [Source: HGNC Symbol; Acc: HGNC: 29853]
5.00	KIAA1211	57482	CRACD	capping protein inhibiting regulator of actin dynamics [Source: HGNC Symbol; Acc: HGNC: 29219]
5.00	MAP3K4	4216	MAP3K4	mitogen-activated protein kinase kinase kinase 4 [Source: HGNC Symbol; Acc: HGNC: 6856]
5.00	VPS13A	23230	VPS13A	vacuolar protein sorting 13 homolog A [Source: HGNC Symbol; Acc: HGNC: 1908]
5.00	XRN1	54464	XRN1	5'-3' exoribonuclease 1 [Source: HGNC Symbol; Acc: HGNC: 30654]
4.98	FLT3	2322	FLT3	fms related tyrosine kinase 3 [Source: HGNC Symbol; Acc: HGNC: 3765]
4.95	ATRX	546	ATRX	ATRX chromatin remodeler [Source: HGNC Symbol; Acc: HGNC: 886]
4.95	FLT1	2321	FLT1	fms related tyrosine kinase 1 [Source: HGNC Symbol; Acc: HGNC: 3763]
4.95	MLL	4297	KMT2A	lysine methyltransferase 2A [Source: HGNC Symbol; Acc: HGNC: 7132]
4.92	MED12	9968	MED12	mediator complex subunit 12 [Source: HGNC Symbol; Acc: HGNC: 11957]
4.84	ARID2	196528	ARID2	AT-rich interaction domain 2 [Source: HGNC Symbol; Acc: HGNC: 18037]
4.84	GATA3	2625	GATA3	GATA binding protein 3 [Source: HGNC Symbol; Acc: HGNC: 4172]
4.84	PIK3CG	5294	PIK3CG	phosphatidylinositol-4,5-bisphosphate 3-kinase catalytic subunit gamma [Source: HGNC Symbol; Acc: HGNC: 8978]
4.80	PIK3CA	5290	PIK3CA	phosphatidylinositol-4,5-bisphosphate 3-kinase catalytic subunit alpha [Source: HGNC Symbol; Acc: HGNC: 8975]
4.76	PARK2	5071	PRKN	parkin RBR E3 ubiquitin protein ligase [Source: HGNC Symbol; Acc: HGNC: 8607]
4.76	PIK3C2G	5288	PIK3C2G	phosphatidylinositol-4-phosphate 3-kinase catalytic subunit type 2 gamma [Source: HGNC Symbol; Acc: HGNC: 8973]
4.52	KDR	3791	KDR	kinase insert domain receptor [Source: HGNC Symbol; Acc: HGNC: 6307]
4.46	CREBBP	1387	CREBBP	CREB binding protein [Source: HGNC Symbol; Acc: HGNC: 2348]
4.46	DNMT3A	1788	DNMT3A	DNA methyltransferase 3 alpha [Source: HGNC Symbol; Acc: HGNC: 2978]
4.37	INSR	3643	INSR	insulin receptor [Source: HGNC Symbol; Acc: HGNC: 6091]
4.35	COL18A1	80781	COL18A1	collagen type XVIII alpha 1 chain [Source: HGNC Symbol; Acc: HGNC: 2195]
4.35	MDN1	23195	MDN1	midasin AAA ATPase 1 [Source: HGNC Symbol; Acc: HGNC: 18302]
4.35	PDGFRA	5156	PDGFRA	platelet derived growth factor receptor alpha [Source: HGNC Symbol; Acc: HGNC: 8803]
4.35	ZDBF2	57683	ZDBF2	zinc finger DBF-type containing 2 [Source: HGNC Symbol; Acc: HGNC: 29313]
4.09	NOTCH2	4853	NOTCH2	notch receptor 2 [Source: HGNC Symbol; Acc: HGNC: 7882]
4.03	NFE2L2	4780	NFE2L2	nuclear factor, erythroid 2 like 2 [Source: HGNC Symbol; Acc: HGNC: 7782]
4.03	AR	367	AR	androgen receptor [Source: HGNC Symbol; Acc: HGNC: 644]

4.03	DICER1	23405	DICER1	dicer 1, ribonuclease III [Source: HGNC Symbol; Acc: HGNC: 17098]
4.03	KDM5C	8242	KDM5C	lysine demethylase 5C [Source: HGNC Symbol; Acc: HGNC: 11114]
4.03	PAX5	5079	PAX5	paired box 5 [Source: HGNC Symbol; Acc: HGNC: 8619]
4.03	TET1	80312	TET1	tet methylcytosine dioxygenase 1 [Source: HGNC Symbol; Acc: HGNC: 29484]
3.96	ROS1	6098	ROS1	ROS proto-oncogene 1, receptor tyrosine kinase [Source: HGNC Symbol; Acc: HGNC: 10261]
3.95	MET	4233	MET	MET proto-oncogene, receptor tyrosine kinase [Source: HGNC Symbol; Acc: HGNC: 7029]
3.82	NCAM2	4685	NCAM2	neural cell adhesion molecule 2 [Source: HGNC Symbol; Acc: HGNC: 7657]
3.81	AMER1	139285	AMER1	APC membrane recruitment protein 1 [Source: HGNC Symbol; Acc: HGNC: 26837]
3.80	RIN3	79890	AL159141.1	Ras and Rab interactor 3 [Source: NCBI gene; Acc: 79890]
3.63	KIT	3815	KIT	KIT proto-oncogene, receptor tyrosine kinase [Source: HGNC Symbol; Acc: HGNC: 6342]
3.62	AHNAK2	113146	AHNAK2	AHNAK nucleoprotein 2 [Source: HGNC Symbol; Acc: HGNC: 20125]
3.62	KIAA0226	9711	RUBCN	rubicon autophagy regulator [Source: HGNC Symbol; Acc: HGNC: 28991]
3.62	OR5D14	219436	OR5D14	olfactory receptor family 5 subfamily D member 14 [Source: HGNC Symbol; Acc: HGNC: 15281]
3.62	VWDE	221806	VWDE	von Willebrand factor D and EGF domains [Source: HGNC Symbol; Acc: HGNC: 21897]
3.56	EGFR	1956	EGFR	epidermal growth factor receptor [Source: HGNC Symbol; Acc: HGNC: 3236]
3.47	IGF1R	3480	IGF1R	insulin like growth factor 1 receptor [Source: HGNC Symbol; Acc: HGNC: 5465]
3.36	PARP1	142	PARP1	poly(ADP-ribose) polymerase 1 [Source: HGNC Symbol; Acc: HGNC: 270]
3.36	SF3B1	23451	SF3B1	splicing factor 3b subunit 1 [Source: HGNC Symbol; Acc: HGNC: 10768]
3.33	APBB1IP	54518	APBB1IP	amyloid beta precursor protein binding family B member 1 interacting protein [Source: HGNC Symbol; Acc: HGNC: 17379]
3.33	ARHGAP4	393	ARHGAP4	Rho GTPase activating protein 4 [Source: HGNC Symbol; Acc: HGNC: 674]
3.33	CEP128	145508	CEP128	centrosomal protein 128 [Source: HGNC Symbol; Acc: HGNC: 20359]
3.33	FPR1	2357	FPR1	formyl peptide receptor 1 [Source: HGNC Symbol; Acc: HGNC: 3826]
3.33	RAD51C	5889	RAD51C	RAD51 paralog C [Source: HGNC Symbol; Acc: HGNC: 9820]
3.33	TP73	7161	TP73	tumor protein p73 [Source: HGNC Symbol; Acc: HGNC: 12003]
3.28	BCOR	54880	BCOR	BCL6 corepressor [Source: HGNC Symbol; Acc: HGNC: 20893]
3.23	BAP1	8314	BAP1	BRCA1 associated protein 1 [Source: HGNC Symbol; Acc: HGNC: 950]
3.23	IRS2	8660	IRS2	insulin receptor substrate 2 [Source: HGNC Symbol; Acc: HGNC: 6126]
3.23	PIK3CD	5293	PIK3CD	phosphatidylinositol-4,5-bisphosphate 3-kinase catalytic subunit delta [Source: HGNC Symbol; Acc: HGNC: 8977]
3.23	WT1	7490	WT1	WT1 transcription factor [Source: HGNC Symbol; Acc: HGNC: 12796]
3.18	RNF213	57674	RNF213	ring finger protein 213 [Source: HGNC Symbol; Acc: HGNC: 14539]
2.97	EPHB1	2047	EPHB1	EPH receptor B1 [Source: HGNC Symbol; Acc: HGNC: 3392]
2.97	FLT4	2324	FLT4	fms related tyrosine kinase 4 [Source: HGNC Symbol; Acc: HGNC: 3767]
2.97	SETD2	29072	SETD2	SET domain containing 2, histone lysine methyltransferase [Source: HGNC Symbol; Acc: HGNC: 18420]
2.97	TSC2	7249	TSC2	TSC complex subunit 2 [Source: HGNC Symbol; Acc: HGNC: 12363]
2.90	ACSM2B	348158	ACSM2B	acyl-CoA synthetase medium chain family member 2B [Source: HGNC Symbol; Acc: HGNC: 30931]

2.90	DCHS1	8642	DCHS1	dachsous cadherin-related 1 [Source: HGNC Symbol; Acc: HGNC: 13681]
2.90	KIF1A	547	KIF1A	kinesin family member 1A [Source: HGNC Symbol; Acc: HGNC: 888]
2.90	MEGF8	1954	MEGF8	multiple EGF like domains 8 [Source: HGNC Symbol; Acc: HGNC: 3233]
2.90	PLEC	5339	PLEC	plectin [Source: HGNC Symbol; Acc: HGNC: 9069]
2.86	INPP4A	3631	INPP4A	inositol polyphosphate-4-phosphatase type I A [Source: HGNC Symbol; Acc: HGNC: 6074]
2.80	HNF1A	6927	HNF1A	HNF1 homeobox A [Source: HGNC Symbol; Acc: HGNC: 11621]
2.80	SMO	6608	SMO	smoothened, frizzled class receptor [Source: HGNC Symbol; Acc: HGNC: 11119]
2.77	ALK	238	ALK	ALK receptor tyrosine kinase [Source: HGNC Symbol; Acc: HGNC: 427]
2.73	ETV6	2120	ETV6	ETS variant transcription factor 6 [Source: HGNC Symbol; Acc: HGNC: 3495]
2.71	ABL1	25	ABL1	ABL proto-oncogene 1, non-receptor tyrosine kinase [Source: HGNC Symbol; Acc: HGNC: 76]
2.53	FANCD2	2177	FANCD2	FA complementation group D2 [Source: HGNC Symbol; Acc: HGNC: 3585]
2.48	EP300	2033	EP300	E1A binding protein p300 [Source: HGNC Symbol; Acc: HGNC: 3373]
2.42	CBL	867	CBL	Cbl proto-oncogene [Source: HGNC Symbol; Acc: HGNC: 1541]
2.42	CCND2	894	CCND2	cyclin D2 [Source: HGNC Symbol; Acc: HGNC: 1583]
2.42	CDC73	79577	CDC73	cell division cycle 73 [Source: HGNC Symbol; Acc: HGNC: 16783]
2.42	CDK12	51755	CDK12	cyclin dependent kinase 12 [Source: HGNC Symbol; Acc: HGNC: 24224]
2.42	ERCC5	2073	ERCC5	ERCC excision repair 5, endonuclease [Source: HGNC Symbol; Acc: HGNC: 3437]
2.42	ETV1	2115	ETV1	ETS variant transcription factor 1 [Source: HGNC Symbol; Acc: HGNC: 3490]
2.42	IKZF1	10320	IKZF1	IKAROS family zinc finger 1 [Source: HGNC Symbol; Acc: HGNC: 13176]
2.42	MDM4	4194	MDM4	MDM4 regulator of p53 [Source: HGNC Symbol; Acc: HGNC: 6974]
2.42	TNFAIP3	7128	TNFAIP3	TNF alpha induced protein 3 [Source: HGNC Symbol; Acc: HGNC: 11896]
2.37	BRAF	673	BRAF	B-Raf proto-oncogene, serine/threonine kinase [Source: HGNC Symbol; Acc: HGNC: 1097]
2.37	ERBB2	2064	ERBB2	erb-b2 receptor tyrosine kinase 2 [Source: HGNC Symbol; Acc: HGNC: 3430]
2.26	FGFR1	2260	FGFR1	fibroblast growth factor receptor 1 [Source: HGNC Symbol; Acc: HGNC: 3688]
2.19	BRCA1	672	BRCA1	BRCA1 DNA repair associated [Source: HGNC Symbol; Acc: HGNC: 1100]
2.19	RICTOR	253260	RICTOR	RPTOR independent companion of MTOR complex 2 [Source: HGNC Symbol; Acc: HGNC: 28611]
2.10	CDKN2A	1029	CDKN2A	cyclin dependent kinase inhibitor 2A [Source: HGNC Symbol; Acc: HGNC: 1787]
1.98	MTOR	2475	MTOR	mechanistic target of rapamycin kinase [Source: HGNC Symbol; Acc: HGNC: 3942]
1.98	NTRK1	4914	NTRK1	neurotrophic receptor tyrosine kinase 1 [Source: HGNC Symbol; Acc: HGNC: 8031]
1.98	TSC1	7248	TSC1	TSC complex subunit 1 [Source: HGNC Symbol; Acc: HGNC: 12362]
1.98	GNAS	2778	GNAS	GNAS complex locus [Source: HGNC Symbol; Acc: HGNC: 4392]
1.91	AKAP9	10142	AKAP9	A-kinase anchoring protein 9 [Source: HGNC Symbol; Acc: HGNC: 379]
1.81	HRAS	3265	HRAS	HRas proto-oncogene, GTPase [Source: HGNC Symbol; Acc: HGNC: 5173]
1.67	ATP1A2	477	ATP1A2	ATPase Na+/K+ transporting subunit alpha 2 [Source: HGNC Symbol; Acc: HGNC: 800]
1.67	EPG5	57724	EPG5	ectopic P-granules autophagy protein 5 homolog [Source: HGNC Symbol; Acc: HGNC: 29331]

1.67	NCOR2	9612	NCOR2	nuclear receptor corepressor 2 [Source: HGNC Symbol; Acc: HGNC: 7673]
1.67	PSMD2	5708	PSMD2	proteasome 26S subunit, non-ATPase 2 [Source: HGNC Symbol; Acc: HGNC: 9559]
1.67	PTGFRN	5738	PTGFRN	prostaglandin F2 receptor inhibitor [Source: HGNC Symbol; Acc: HGNC: 9601]
1.67	RBL1	5933	RBL1	RB transcriptional corepressor like 1 [Source: HGNC Symbol; Acc: HGNC: 9893]
1.67	SPATA6	54558	SPATA6	spermatogenesis associated 6 [Source: HGNC Symbol; Acc: HGNC: 18309]
1.67	TRAPPC9	83696	TRAPPC9	trafficking protein particle complex 9 [Source: HGNC Symbol; Acc: HGNC: 30832]
1.67	YTHDC2	64848	YTHDC2	YTH domain containing 2 [Source: HGNC Symbol; Acc: HGNC: 24721]
1.61	CIC	23152	CIC	capicua transcriptional repressor [Source: HGNC Symbol; Acc: HGNC: 14214]
1.61	PALB2	79728	PALB2	partner and localizer of BRCA2 [Source: HGNC Symbol; Acc: HGNC: 26144]
1.61	PTCH1	5727	PTCH1	patched 1 [Source: HGNC Symbol; Acc: HGNC: 9585]
1.52	SOX2	6657	SOX2	SRY-box transcription factor 2 [Source: HGNC Symbol; Acc: HGNC: 11195]
1.49	AKT3	10000	AKT3	AKT serine/threonine kinase 3 [Source: HGNC Symbol; Acc: HGNC: 393]
1.45	CHD7	55636	CHD7	chromodomain helicase DNA binding protein 7 [Source: HGNC Symbol; Acc: HGNC: 20626]
1.45	CLTCL1	8218	CLTCL1	clathrin heavy chain like 1 [Source: HGNC Symbol; Acc: HGNC: 2093]
1.45	MUC12	10071	MUC12	mucin 12, cell surface associated [Source: HGNC Symbol; Acc: HGNC: 7510]
1.45	RGS7	6000	RGS7	regulator of G protein signaling 7 [Source: HGNC Symbol; Acc: HGNC: 10003]
1.45	ZNF208	7757	ZNF208	zinc finger protein 208 [Source: HGNC Symbol; Acc: HGNC: 12999]
1.36	JAK2	3717	JAK2	Janus kinase 2 [Source: HGNC Symbol; Acc: HGNC: 6192]
1.36	RET	5979	RET	ret proto-oncogene [Source: HGNC Symbol; Acc: HGNC: 9967]
1.27	TDRD7	23424	TDRD7	tudor domain containing 7 [Source: HGNC Symbol; Acc: HGNC: 30831]
1.14	SMAD4	4089	SMAD4	SMAD family member 4 [Source: HGNC Symbol; Acc: HGNC: 6770]

Table S4. Genes Frequently mutated in Small Cell Lung Carcinomas (SCLCs).

Mutation Rate %	Original	Entrez	Gene	Gene
92.76	TP53	7157	TP53	tumor protein p53 [Source: HGNC Symbol; Acc: HGNC: 11998]
66.67	LRP1B	53353	LRP1B	LDL receptor related protein 1B [Source: HGNC Symbol; Acc: HGNC: 6693]
60.18	RB1	5925	RB1	RB transcriptional corepressor 1 [Source: HGNC Symbol; Acc: HGNC: 9884]
55.26	CSMD3	114788	CSMD3	CUB and Sushi multiple domains 3 [Source: HGNC Symbol; Acc: HGNC: 19291]
33.50	ZFHX4	79776	ZFHX4	zinc finger homeobox 4 [Source: HGNC Symbol; Acc: HGNC: 30939]
33.33	SYNE1	23345	SYNE1	spectrin repeat containing nuclear envelope protein 1 [Source: HGNC Symbol; Acc: HGNC: 17089]
29.00	USH2A	7399	USH2A	usherin [Source: HGNC Symbol; Acc: HGNC: 12601]
23.68	SPHKAP	80309	SPHKAP	SPHK1 interactor, AKAP domain containing [Source: HGNC Symbol; Acc: HGNC: 30619]
21.08	MLL2	8085	KMT2D	lysine methyltransferase 2D [Source: HGNC Symbol; Acc: HGNC: 7133]
19.09	KIAA1211	57482	CRACD	capping protein inhibiting regulator of actin dynamics [Source: HGNC Symbol; Acc: HGNC: 29219]
18.50	COL11A1	1301	COL11A1	collagen type XI alpha 1 chain [Source: HGNC Symbol; Acc: HGNC: 2186]
18.18	FMN2	56776	FMN2	formin 2 [Source: HGNC Symbol; Acc: HGNC: 14074]

16.36	ADAMTS 12	81792	ADAMTS12	ADAM metallopeptidase with thrombospondin type 1 motif 12 [Source: HGNC Symbol; Acc: HGNC: 14605]
15.79	DST	667	DST	dystonin [Source: HGNC Symbol; Acc: HGNC: 1090]
15.20	PCLO	27445	PCLO	piccolo presynaptic cytomatrix protein [Source: HGNC Symbol; Acc: HGNC: 13406]
14.22	NCAM2	4685	NCAM2	neural cell adhesion molecule 2 [Source: HGNC Symbol; Acc: HGNC: 7657]
14.04	THSD7B	80731	THSD7B	thrombospondin type 1 domain containing 7B [Source: HGNC Symbol; Acc: HGNC: 29348]
12.73	EPHA5	2044	EPHA5	EPH receptor A5 [Source: HGNC Symbol; Acc: HGNC: 3389]
11.82	ALMS1	7840	ALMS1	ALMS1 centrosome and basal body associated protein [Source: HGNC Symbol; Acc: HGNC: 428]
11.50	COL22A1	169044	COL22A1	collagen type XXII alpha 1 chain [Source: HGNC Symbol; Acc: HGNC: 22989]
11.40	PTPRD	5789	PTPRD	protein tyrosine phosphatase receptor type D [Source: HGNC Symbol; Acc: HGNC: 9668]
11.40	PTPRT	11122	PTPRT	protein tyrosine phosphatase receptor type T [Source: HGNC Symbol; Acc: HGNC: 9682]
10.50	LAMA1	284217	LAMA1	laminin subunit alpha 1 [Source: HGNC Symbol; Acc: HGNC: 6481]
10.50	LAMA2	3908	LAMA2	laminin subunit alpha 2 [Source: HGNC Symbol; Acc: HGNC: 6482]
10.50	PLEC	5339	PLEC	plectin [Source: HGNC Symbol; Acc: HGNC: 9069]
10.50	ZNF208	7757	ZNF208	zinc finger protein 208 [Source: HGNC Symbol; Acc: HGNC: 12999]
10.29	MLL3	58508	KMT2C	lysine methyltransferase 2C [Source: HGNC Symbol; Acc: HGNC: 13726]
10.00	ASPM	259266	ASPM	abnormal spindle microtubule assembly [Source: HGNC Symbol; Acc: HGNC: 19048]
10.00	DNAH9	1770	DNAH9	dynein axonemal heavy chain 9 [Source: HGNC Symbol; Acc: HGNC: 2953]
10.00	STAB2	55576	STAB2	stabilin 2 [Source: HGNC Symbol; Acc: HGNC: 18629]
9.50	NOTCH1	4851	NOTCH1	notch receptor 1 [Source: HGNC Symbol; Acc: HGNC: 7881]
9.09	GRIN2A	2903	GRIN2A	glutamate ionotropic receptor NMDA type subunit 2A [Source: HGNC Symbol; Acc: HGNC: 4585]
9.00	FAT1	2195	FAT1	FAT atypical cadherin 1 [Source: HGNC Symbol; Acc: HGNC: 3595]
8.50	CHD7	55636	CHD7	chromodomain helicase DNA binding protein 7 [Source: HGNC Symbol; Acc: HGNC: 20626]
8.33	ZNF521	25925	ZNF521	zinc finger protein 521 [Source: HGNC Symbol; Acc: HGNC: 24605]
8.18	XRN1	54464	XRN1	5'-3' exoribonuclease 1 [Source: HGNC Symbol; Acc: HGNC: 30654]
8.00	AHNAK2	113146	AHNAK2	AHNAK nucleoprotein 2 [Source: HGNC Symbol; Acc: HGNC: 20125]
8.00	GPR98	84059	ADGRV1	adhesion G protein-coupled receptor V1 [Source: HGNC Symbol; Acc: HGNC: 17416]
8.00	LRFN5	145581	LRFN5	leucine rich repeat and fibronectin type III domain containing 5 [Source: HGNC Symbol; Acc: HGNC: 20360]
7.84	CREBBP	1387	CREBBP	CREB binding protein [Source: HGNC Symbol; Acc: HGNC: 2348]
7.50	NOTCH3	4854	NOTCH3	notch receptor 3 [Source: HGNC Symbol; Acc: HGNC: 7883]
7.35	EP300	2033	EP300	E1A binding protein p300 [Source: HGNC Symbol; Acc: HGNC: 3373]
7.27	BSN	8927	BSN	bassoon presynaptic cytomatrix protein [Source: HGNC Symbol; Acc: HGNC: 1117]
7.27	HECW2	57520	HECW2	HECT, C2 and WW domain containing E3 ubiquitin protein ligase 2 [Source: HGNC Symbol; Acc: HGNC: 29853]
7.27	PIK3C2G	5288	PIK3C2G	phosphatidylinositol-4-phosphate 3-kinase catalytic subunit type 2 gamma [Source: HGNC Symbol; Acc: HGNC: 8973]
7.02	DSCAML1	57453	DSCAML1	DS cell adhesion molecule like 1 [Source: HGNC Symbol; Acc: HGNC: 14656]
7.02	PIK3CG	5294	PIK3CG	phosphatidylinositol-4,5-bisphosphate 3-kinase catalytic subunit gamma [Source: HGNC Symbol; Acc: HGNC: 8978]

7.02	PTPRZ1	5803	PTPRZ1	protein tyrosine phosphatase receptor type Z1 [Source: HGNC Symbol; Acc: HGNC: 9685]
7.00	DCHS1	8642	DCHS1	dachsous cadherin-related 1 [Source: HGNC Symbol; Acc: HGNC: 13681]
7.00	KIF1A	547	KIF1A	kinesin family member 1A [Source: HGNC Symbol; Acc: HGNC: 888]
6.37	PDE4DIP	9659	PDE4DIP	phosphodiesterase 4D interacting protein [Source: HGNC Symbol; Acc: HGNC: 15580]
6.36	COL5A3	50509	COL5A3	collagen type V alpha 3 chain [Source: HGNC Symbol; Acc: HGNC: 14864]
6.36	FPR1	2357	FPR1	formyl peptide receptor 1 [Source: HGNC Symbol; Acc: HGNC: 3826]
6.36	TRAPPC9	83696	TRAPPC9	trafficking protein particle complex 9 [Source: HGNC Symbol; Acc: HGNC: 30832]
6.36	VPS13A	23230	VPS13A	vacuolar protein sorting 13 homolog A [Source: HGNC Symbol; Acc: HGNC: 1908]
6.33	ERBB4	2066	ERBB4	erb-b2 receptor tyrosine kinase 4 [Source: HGNC Symbol; Acc: HGNC: 3432]
6.33	PIK3CA	5290	PIK3CA	phosphatidylinositol-4,5-bisphosphate 3-kinase catalytic subunit alpha [Source: HGNC Symbol; Acc: HGNC: 8975]
6.00	CNTNAP2	26047	CNTNAP2	contactin associated protein like 2 [Source: HGNC Symbol; Acc: HGNC: 13830]
6.00	OR5D14	219436	OR5D14	olfactory receptor family 5 subfamily D member 14 [Source: HGNC Symbol; Acc: HGNC: 15281]
6.00	RGS7	6000	RGS7	regulator of G protein signaling 7 [Source: HGNC Symbol; Acc: HGNC: 10003]
5.88	ATRX	546	ATRX	ATRX chromatin remodeler [Source: HGNC Symbol; Acc: HGNC: 886]
5.50	MED12	9968	MED12	mediator complex subunit 12 [Source: HGNC Symbol; Acc: HGNC: 11957]
5.50	MUC12	10071	MUC12	mucin 12, cell surface associated [Source: HGNC Symbol; Acc: HGNC: 7510]
5.50	PRIC285	85441	HELZ2	helicase with zinc finger 2 [Source: HGNC Symbol; Acc: HGNC: 30021]
5.50	VWDE	221806	VWDE	von Willebrand factor D and EGF domains [Source: HGNC Symbol; Acc: HGNC: 21897]
5.45	ARHGAP4	393	ARHGAP4	Rho GTPase activating protein 4 [Source: HGNC Symbol; Acc: HGNC: 674]
5.45	ATP1A2	477	ATP1A2	ATPase Na+/K+ transporting subunit alpha 2 [Source: HGNC Symbol; Acc: HGNC: 800]
5.45	CEP128	145508	CEP128	centrosomal protein 128 [Source: HGNC Symbol; Acc: HGNC: 20359]
5.45	EPG5	57724	EPG5	ectopic P-granules autophagy protein 5 homolog [Source: HGNC Symbol; Acc: HGNC: 29331]
5.45	KIAA1324L	222223	KIAA1324L	KIAA1324 like [Source: HGNC Symbol; Acc: HGNC: 21945]
5.45	MAP3K4	4216	MAP3K4	mitogen-activated protein kinase kinase kinase 4 [Source: HGNC Symbol; Acc: HGNC: 6856]
5.45	PKD1	5310	PKD1	polycystin 1, transient receptor potential channel interacting [Source: HGNC Symbol; Acc: HGNC: 9008]
5.45	SMARCA2	6595	SMARCA2	SWI/SNF related, matrix associated, actin dependent regulator of chromatin, subfamily a, member 2 [Source: HGNC Symbol; Acc: HGNC: 11098]
5.45	SORL1	6653	SORL1	sortilin related receptor 1 [Source: HGNC Symbol; Acc: HGNC: 11185]
5.45	STAG2	10735	STAG2	stromal antigen 2 [Source: HGNC Symbol; Acc: HGNC: 11355]
5.45	TP73	7161	TP73	tumor protein p73 [Source: HGNC Symbol; Acc: HGNC: 12003]
5.43	PTEN	5728	PTEN	phosphatase and tensin homolog [Source: HGNC Symbol; Acc: HGNC: 9588]
4.98	ATM	472	ATM	ATM serine/threonine kinase [Source: HGNC Symbol; Acc: HGNC: 795]
4.98	RET	5979	RET	ret proto-oncogene [Source: HGNC Symbol; Acc: HGNC: 9967]
4.90	EPHB1	2047	EPHB1	EPH receptor B1 [Source: HGNC Symbol; Acc: HGNC: 3392]
4.58	APC	324	APC	APC regulator of WNT signaling pathway [Source: HGNC Symbol; Acc: HGNC: 583]

4.55	ADAMTS 2	9509	ADAMTS2	ADAM metallopeptidase with thrombospondin type 1 motif 2 [Source: HGNC Symbol; Acc: HGNC: 218]
4.55	APBB1IP	54518	APBB1IP	amyloid beta precursor protein binding family B member 1 interacting protein [Source: HGNC Symbol; Acc: HGNC: 17379]
4.55	EIF4G3	8672	EIF4G3	eukaryotic translation initiation factor 4 gamma 3 [Source: HGNC Symbol; Acc: HGNC: 3298]
4.55	FAP	2191	FAP	fibroblast activation protein alpha [Source: HGNC Symbol; Acc: HGNC: 3590]
4.55	NCOR2	9612	NCOR2	nuclear receptor corepressor 2 [Source: HGNC Symbol; Acc: HGNC: 7673]
4.55	POLE	5426	POLE	DNA polymerase epsilon, catalytic subunit [Source: HGNC Symbol; Acc: HGNC: 9177]
4.55	PSMD2	5708	PSMD2	proteasome 26S subunit, non-ATPase 2 [Source: HGNC Symbol; Acc: HGNC: 9559]
4.55	PTGFRN	5738	PTGFRN	prostaglandin F2 receptor inhibitor [Source: HGNC Symbol; Acc: HGNC: 9601]
4.55	SPATA6	54558	SPATA6	spermatogenesis associated 6 [Source: HGNC Symbol; Acc: HGNC: 18309]
4.52	ALK	238	ALK	ALK receptor tyrosine kinase [Source: HGNC Symbol; Acc: HGNC: 427]
4.41	NF1	4763	NF1	neurofibromin 1 [Source: HGNC Symbol; Acc: HGNC: 7765]
4.41	NOTCH2	4853	NOTCH2	notch receptor 2 [Source: HGNC Symbol; Acc: HGNC: 7882]
4.41	ROS1	6098	ROS1	ROS proto-oncogene 1, receptor tyrosine kinase [Source: HGNC Symbol; Acc: HGNC: 10261]
4.39	AR	367	AR	androgen receptor [Source: HGNC Symbol; Acc: HGNC: 644]
4.39	CBL	867	CBL	Cbl proto-oncogene [Source: HGNC Symbol; Acc: HGNC: 1541]
4.39	NOTCH4	4855	NOTCH4	notch receptor 4 [Source: HGNC Symbol; Acc: HGNC: 7884]
4.39	WT1	7490	WT1	WT1 transcription factor [Source: HGNC Symbol; Acc: HGNC: 12796]
4.00	ACSM2B	348158	ACSM2B	acyl-CoA synthetase medium chain family member 2B [Source: HGNC Symbol; Acc: HGNC: 30931]
4.00	MDN1	23195	MDN1	midasin AAA ATPase 1 [Source: HGNC Symbol; Acc: HGNC: 18302]
4.00	MEGF8	1954	MEGF8	multiple EGF like domains 8 [Source: HGNC Symbol; Acc: HGNC: 3233]
3.92	AKAP9	10142	AKAP9	A-kinase anchoring protein 9 [Source: HGNC Symbol; Acc: HGNC: 379]
3.69	KIT	3815	KIT	KIT proto-oncogene, receptor tyrosine kinase [Source: HGNC Symbol; Acc: HGNC: 6342]
3.64	RBL1	5933	RBL1	RB transcriptional corepressor like 1 [Source: HGNC Symbol; Acc: HGNC: 9893]
3.64	YTHDC2	64848	YTHDC2	YTH domain containing 2 [Source: HGNC Symbol; Acc: HGNC: 24721]
3.62	EGFR	1956	EGFR	epidermal growth factor receptor [Source: HGNC Symbol; Acc: HGNC: 3236]
3.62	KDR	3791	KDR	kinase insert domain receptor [Source: HGNC Symbol; Acc: HGNC: 6307]
3.62	PDGFRA	5156	PDGFRA	platelet derived growth factor receptor alpha [Source: HGNC Symbol; Acc: HGNC: 8803]
3.51	IL7R	3575	IL7R	interleukin 7 receptor [Source: HGNC Symbol; Acc: HGNC: 6024]
3.51	KEAP1	9817	KEAP1	kelch like ECH associated protein 1 [Source: HGNC Symbol; Acc: HGNC: 23177]
3.50	COL18A1	80781	COL18A1	collagen type XVIII alpha 1 chain [Source: HGNC Symbol; Acc: HGNC: 2195]
3.43	NTRK1	4914	NTRK1	neurotrophic receptor tyrosine kinase 1 [Source: HGNC Symbol; Acc: HGNC: 8031]
3.43	TSC2	7249	TSC2	TSC complex subunit 2 [Source: HGNC Symbol; Acc: HGNC: 12363]
3.17	GNAS	2778	GNAS	GNAS complex locus [Source: HGNC Symbol; Acc: HGNC: 4392]
3.00	CLTCL1	8218	CLTCL1	clathrin heavy chain like 1 [Source: HGNC Symbol; Acc: HGNC: 2093]
3.00	RBL2	5934	RBL2	RB transcriptional corepressor like 2 [Source: HGNC Symbol; Acc: HGNC: 9894]
3.00	SALL1	6299	SALL1	spalt like transcription factor 1 [Source: HGNC Symbol; Acc: HGNC: 10524]

2.94	ARID1A	8289	ARID1A	AT-rich interaction domain 1A [Source: HGNC Symbol; Acc: HGNC: 11110]
2.94	FLT1	2321	FLT1	fms related tyrosine kinase 1 [Source: HGNC Symbol; Acc: HGNC: 3763]
2.94	MLL	4297	KMT2A	lysine methyltransferase 2A [Source: HGNC Symbol; Acc: HGNC: 7132]
2.94	NUP98	4928	NUP98	nucleoporin 98 [Source: HGNC Symbol; Acc: HGNC: 8068]
2.94	SMARCA 4	6597	SMARCA4	SWI/SNF related, matrix associated, actin dependent regulator of chromatin, subfamily a, member 4 [Source: HGNC Symbol; Acc: HGNC: 11100]
2.94	TSHR	7253	TSHR	thyroid stimulating hormone receptor [Source: HGNC Symbol; Acc: HGNC: 12373]
2.73	GAS7	8522	AC005747.1	growth arrest specific 7 [Source: NCBI gene; Acc: 8522]
2.73	INPP4A	3631	INPP4A	inositol polyphosphate-4-phosphatase type I A [Source: HGNC Symbol; Acc: HGNC: 6074]
2.73	IRS1	3667	IRS1	insulin receptor substrate 1 [Source: HGNC Symbol; Acc: HGNC: 6125]
2.73	PAK7	57144	PAK5	p21 (RAC1) activated kinase 5 [Source: HGNC Symbol; Acc: HGNC: 15916]
2.63	EPHA3	2042	EPHA3	EPH receptor A3 [Source: HGNC Symbol; Acc: HGNC: 3387]
2.63	GATA3	2625	GATA3	GATA binding protein 3 [Source: HGNC Symbol; Acc: HGNC: 4172]
2.63	IKZF1	10320	IKZF1	IKAROS family zinc finger 1 [Source: HGNC Symbol; Acc: HGNC: 13176]
2.63	PALB2	79728	PALB2	partner and localizer of BRCA2 [Source: HGNC Symbol; Acc: HGNC: 26144]
2.63	PAX5	5079	PAX5	paired box 5 [Source: HGNC Symbol; Acc: HGNC: 8619]
2.63	PTCH1	5727	PTCH1	patched 1 [Source: HGNC Symbol; Acc: HGNC: 9585]
2.63	TET1	80312	TET1	tet methylcytosine dioxygenase 1 [Source: HGNC Symbol; Acc: HGNC: 29484]
2.50	BCOR	54880	BCOR	BCL6 corepressor [Source: HGNC Symbol; Acc: HGNC: 20893]
2.50	INSR	3643	INSR	insulin receptor [Source: HGNC Symbol; Acc: HGNC: 6091]
2.50	MSH3	4437	MSH3	mutS homolog 3 [Source: HGNC Symbol; Acc: HGNC: 7326]
2.50	ZDBF2	57683	ZDBF2	zinc finger DBF-type containing 2 [Source: HGNC Symbol; Acc: HGNC: 29313]
2.50	ZNF195	7748	ZNF195	zinc finger protein 195 [Source: HGNC Symbol; Acc: HGNC: 12986]
2.45	DNMT3A	1788	DNMT3A	DNA methyltransferase 3 alpha [Source: HGNC Symbol; Acc: HGNC: 2978]
2.45	FLT4	2324	FLT4	fms related tyrosine kinase 4 [Source: HGNC Symbol; Acc: HGNC: 3767]
2.45	RNF213	57674	RNF213	ring finger protein 213 [Source: HGNC Symbol; Acc: HGNC: 14539]
2.29	CDKN2A	1029	CDKN2A	cyclin dependent kinase inhibitor 2A [Source: HGNC Symbol; Acc: HGNC: 1787]
2.29	IDH1	3417	IDH1	isocitrate dehydrogenase (NADP(+)) 1 [Source: HGNC Symbol; Acc: HGNC: 5382]
2.26	ERBB2	2064	ERBB2	erb-b2 receptor tyrosine kinase 2 [Source: HGNC Symbol; Acc: HGNC: 3430]
1.96	MTOR	2475	MTOR	mechanistic target of rapamycin kinase [Source: HGNC Symbol; Acc: HGNC: 3942]
1.96	NTRK3	4916	NTRK3	neurotrophic receptor tyrosine kinase 3 [Source: HGNC Symbol; Acc: HGNC: 8033]
1.96	PDGFRB	5159	PDGFRB	platelet derived growth factor receptor beta [Source: HGNC Symbol; Acc: HGNC: 8804]
1.82	NTM	50863	NTM	neurotrimin [Source: HGNC Symbol; Acc: HGNC: 17941]
1.82	SF3B1	23451	SF3B1	splicing factor 3b subunit 1 [Source: HGNC Symbol; Acc: HGNC: 10768]
1.81	ABL1	25	ABL1	ABL proto-oncogene 1, non-receptor tyrosine kinase [Source: HGNC Symbol; Acc: HGNC: 76]
1.81	JAK3	3718	JAK3	Janus kinase 3 [Source: HGNC Symbol; Acc: HGNC: 6193]
1.75	CDK12	51755	CDK12	cyclin dependent kinase 12 [Source: HGNC Symbol; Acc: HGNC: 24224]
1.75	CIC	23152	CIC	capicua transcriptional repressor [Source: HGNC Symbol; Acc: HGNC: 14214]

1.75	IRS2	8660	IRS2	insulin receptor substrate 2 [Source: HGNC Symbol; Acc: HGNC: 6126]
1.75	KAT6B	23522	KAT6B	lysine acetyltransferase 6B [Source: HGNC Symbol; Acc: HGNC: 17582]
1.75	TDRD7	23424	TDRD7	tudor domain containing 7 [Source: HGNC Symbol; Acc: HGNC: 30831]
1.75	TNFAIP3	7128	TNFAIP3	TNF alpha induced protein 3 [Source: HGNC Symbol; Acc: HGNC: 11896]
1.53	HNF1A	6927	HNF1A	HNF1 homeobox A [Source: HGNC Symbol; Acc: HGNC: 11621]
1.53	SMAD4	4089	SMAD4	SMAD family member 4 [Source: HGNC Symbol; Acc: HGNC: 6770]
1.53	SMO	6608	SMO	smoothened, frizzled class receptor [Source: HGNC Symbol; Acc: HGNC: 11119]
1.50	ETV6	2120	ETV6	ETS variant transcription factor 6 [Source: HGNC Symbol; Acc: HGNC: 3495]
1.50	KIAA0226	9711	RUBCN	rubicon autophagy regulator [Source: HGNC Symbol; Acc: HGNC: 28991]
1.50	RICTOR	253260	RICTOR	RPTOR independent companion of MTOR complex 2 [Source: HGNC Symbol; Acc: HGNC: 28611]
1.47	IGF1R	3480	IGF1R	insulin like growth factor 1 receptor [Source: HGNC Symbol; Acc: HGNC: 5465]
1.47	SETD2	29072	SETD2	SET domain containing 2, histone lysine methyltransferase [Source: HGNC Symbol; Acc: HGNC: 18420]
1.36	FLT3	2322	FLT3	fms related tyrosine kinase 3 [Source: HGNC Symbol; Acc: HGNC: 3765]
1.36	JAK2	3717	JAK2	Janus kinase 2 [Source: HGNC Symbol; Acc: HGNC: 6192]

Table S5. Top 10 recurrently mutated genes among WHO histotypes.

**Gene**	**All**	**NETs**		**NECs**		***p*-Value***	***p*-Value#**
		**TCs**	**ACs**	**LCNECs**	**SCLCs**		
Top10TCsRecurrentlyMutatedGenes							
EIF1AX	7/256(2.73)	3/62(4.84)	4/24(16.66)	0/60(0.0)	0/110(0.0)	0.0001	0.0004
ARID1A	38/582(6.52)	4/85(4.71)	7/73(9.59)	21/220(9.54)	6/204(2.94)	0.02	0.85
LRP1B	118/301(39.20)	3/69(4.34)	0/39(0.0)	39/79(49.36)	76/114(66.67)	<0.0001	<0.0001
NF1	31/543(5.70)	3/85(3.53)	1/52(1.92)	18/202(8.91)	9/204(4.41)	0.12	0.13
DSCAML1	16/301(5.31)	2/69(2.90)	2/39(5.12)	4/79(5.06)	8/114(7.01)	0.71	0.43
DST	28/301(9.30)	2/69(2.90)	0/39(0.0)	8/79(10.12)	18/114(15.78)	0.003	0.0006
FANCD2	4/301(1.32)	2/69(2.90)	0/39(0.0)	2/79(2.53)	0/114(0.0)	0.21	0.62
PCLO	43/469(9.16)	2/69(2.90)	2/39(5.12)	8/157(5.09)	31/204(15.19)	0.001	0.02
ERBB2	16/670(2.38)	3/123(2.43)	2/73(2.73)	6/253(2.37)	5/221(2.26)	1.00	0.79
BAP1	6/375(1.60)	2/85(2.35)	0/52(0.0)	4/124(3.22)	0/114(0.0)	0.17	1.00
Top10ACsRecurrentlyMutatedGenes							
MEN1	28/414(6.76)	1/85(1.17)	18/73(24.66)	9/142(6.33)	0/114(0.0)	<0.0001	0.001
ATP1A2	10/227(4.40)	1/46(2.17)	2/11(18.18)	1/60(1.66)	6/110(5.45)	0.10	0.71
EIF1AX	7/256(2.73)	3/62(4.84)	4/24(16.66)	0/60(0.0)	0/110(0.0)	0.0001	0.0004
SPHKAP	37/301(12.29)	1/69(1.44)	5/39(12.82)	4/79(5.06)	27/114(23.68)	<0.0001	0.009
ARID1A	38/582(6.52)	4/85(4.71)	7/73(9.59)	21/220(9.54)	6/204(2.94)	0.02	0.85
SMARCA4	31/582(5.32)	0/85(0.0)	7/73(9.59)	18/220(8.18)	6/204(2.94)	0.002	0.68
PKD1	11/227(4.84)	0/46(0.0)	1/11(9.09)	4/60(6.66)	6/110(5.45)	0.21	0.30
AMER1	6/301(1.99)	0/62(0.0)	2/24(8.33)	4/105(3.80)	0/110(0.0)	0.01	1.00
RAD51C	5/256(1.95)	1/62(1.61)	2/24(8.33)	2/60(3.33)	0/110(0.0)	0.02	0.34
TDRD7	7/301(2.32)	1/69(1.44)	3/39(7.69)	1/79(1.26)	2/114(1.75)	0.15	0.25
Top10LCNECsRecurrentlyMutatedGenes							
TP53	516/788(65.48)	1/123(0.81)	5/94(5.31)	305/350(87.14)	205/221(92.76)	<0.0001	<0.0001
LRP1B	118/301(39.20)	3/69(4.34)	0/39(0.0)	39/79(49.36)	76/114(66.67)	<0.0001	<0.0001
CSMD3	103/301(34.21)	1/69(1.44)	2/39(5.12)	37/79(46.83)	63/114(55.26)	<0.0001	<0.0001
RB1	248/731(33.92)	1/102(0.98)	2/90(2.22)	112/318(35.22)	133/221(60.18)	<0.0001	<0.0001
SYNE1	60/301(19.93)	1/69(1.44)	0/39(0.0)	21/79(26.58)	38/114(33.33)	<0.0001	<0.0001
ADAMTS12	31/227(13.65)	0/46(0.0)	0/11(0.0)	13/60(21.66)	18/110(16.36)	0.001	<0.0001
USH2A	86/395(21.76)	0/46(0.0)	0/11(0.0)	28/138(20.28)	58/200(29)	<0.0001	<0.0001
STK11	54/607(8.90)	0/114(0.0)	0/90(0.0)	53/272(19.49)	1/131(0.76)	<0.0001	<0.0001
KEAP1	48/480(10)	0/76(0.0)	0/69(0.0)	44/221(19.91)	4/114(3.50)	<0.0001	<0.0001
DNAH9	46/395(11.64)	0/46(0.0)	0/11(0.0)	26/138(18.84)	20/200(10)	0.001	0.0006
Top10SCLCsRecurrentlyMutatedGenes							

TP53	516/788(65.48)	1/123(0.81)	5/94(5.31)	305/350(87.14)	205/221(92.76)	<0.0001	<0.0001
LRP1B	118/301(39.20)	3/69(4.34)	0/39(0.0)	39/79(49.36)	76/114(66.67)	<0.0001	<0.0001
RB1	248/731(33.92)	1/102(0.98)	2/90(2.22)	112/318(35.22)	133/221(60.18)	<0.0001	<0.0001
CSMD3	103/301(34.21)	1/69(1.44)	2/39(5.12)	37/79(46.83)	63/114(55.26)	<0.0001	<0.0001
ZFHX4	82/395(20.75)	0/46(0.0)	0/11(0.0)	15/138(10.86)	67/200(33.50)	<0.0001	<0.0001
SYNE1	60/301(19.93)	1/69(1.44)	0/39(0.0)	21/79(26.58)	38/114(33.33)	<0.0001	<0.0001
USH2A	86/395(21.76)	0/46(0.0)	0/11(0.0)	28/138(20.28)	58/200(29)	<0.0001	<0.0001
SPHKAP	37/301(12.29)	1/69(1.44)	5/39(12.82)	4/79(5.06)	27/114(23.68)	<0.0001	0.009
KMT2D	68/582(11.68)	0/85(0.0)	4/73(5.48)	21/220(9.54)	43/204(21.07)	<0.0001	<0.0001
KIAA1211	24/227(10.57)	0/46(0.0)	0/11(0.0)	3/60(5)	21/110(19.09)	0.0004	0.0009

* *p*-value based on Fisher's exact for categorical variables between four WHO histological group; # *p*-value based on Fisher's exact for categorical variables between NET and NECs.

© 2020 by the authors. Licensee MDPI, Basel, Switzerland. This article is an open access article distributed under the terms and conditions of the Creative Commons Attribution (CC BY) license (http://creativecommons.org/licenses/by/4.0/).
